# Meta-analytic Gaussian Network Aggregation

**DOI:** 10.1007/s11336-021-09764-3

**Published:** 2021-07-15

**Authors:** Sacha Epskamp, Adela-Maria Isvoranu, Mike W.-L. Cheung

**Affiliations:** 1 Department of Psychology, University of Amsterdam, Amsterdam, Netherlands; 2 Centre for Urban Mental Health, University of Amsterdam, Amsterdam, Netherlands; 3 Department of Psychology, National University of Singapore, Singapore, Singapore

**Keywords:** meta-Analysis, network psychometrics, gaussian graphical model

## Abstract

A growing number of publications focus on estimating Gaussian graphical models (GGM, networks of partial correlation coefficients). At the same time, generalizibility and replicability of these highly parameterized models are debated, and sample sizes typically found in datasets may not be sufficient for estimating the underlying network structure. In addition, while recent work emerged that aims to compare networks based on different samples, these studies do not take potential cross-study heterogeneity into account. To this end, this paper introduces methods for estimating GGMs by aggregating over multiple datasets. We first introduce a general maximum likelihood estimation modeling framework in which all discussed models are embedded. This modeling framework is subsequently used to introduce meta-analytic Gaussian network aggregation (MAGNA). We discuss two variants: fixed-effects MAGNA, in which heterogeneity across studies is not taken into account, and random-effects MAGNA, which models sample correlations and takes heterogeneity into account. We assess the performance of MAGNA in large-scale simulation studies. Finally, we exemplify the method using four datasets of post-traumatic stress disorder (PTSD) symptoms, and summarize findings from a larger meta-analysis of PTSD symptom.

**Supplementary Information:**

The online version supplementary material available at 10.1007/s11336-021-09764-3.

The estimation of Gaussian graphical models (GGM; Epskamp et al. 2018; Lauritzen 1996)—network models with nodes representing observed items and edges (links) representing partial correlation coefficients—has gained popularity in recent psychological research (Fried et al. 2017). A recent review indicated that, by the end of 2019, 141 studies in psychopathology have been published in which cross-sectional datasets were analyzed using network models, the majority of which used GGMs (Robinaugh et al. 2020). These studies include high impact studies in diverse research fields, including post-traumatic stress disorder (PTSD; Mcnally et al. 2015), psychosis (Isvoranu et al. 2019), depression (Fried et al. 2016), and personality research (Costantini et al. 2015). The field of Network Psychometrics is concerned with the estimation of such network models from data (Marsman et al. 2018). A growing issue of debate in this field relates to the replicability and generalizability of these results (Forbes et al. 2017; Fried et al. 2018), especially given that datasets used to estimate GGMs are typically relatively small (e.g., hundreds of cases compared to hundreds of parameters). High-dimensional exploratory model estimation may be too ambitious from single datasets with relatively small sample sizes. As such, there is a distinct need for utilizing multiple studies in estimating GGMs. This paper introduces methods for aggregating results across different studies through introducing multi-dataset^1^ GGM models as well as fixed and random-effects meta-analytic GGM estimation. In doing so, this paper also introduces novel extensions for GGMs estimated from single datasets, including methods for imposing equality constraints across parameters as well as analytic derivatives for fitting confirmatory network models and assessing significance of individual parameters.

As raw data often cannot be shared, a method for studying multiple datasets should be able to utilize summary statistics. More precisely, the methods should allow for the analysis of sample correlation matrices, as these are commonly used when estimating GGMs and as different datasets can include measures of the same variables on different measurement scales. Let $\pmb {P}$ represent the population correlation matrix and $\pmb {R}$ the sample correlation matrix—the *maximum likelihood* estimate explained further in Sect. 3.3—of a particular dataset. Epskamp et al. (2017) propose to model the GGM through the following equation:^2^

1 $$\begin{aligned} \pmb {P} = \pmb {\Delta } \left( \pmb {I} - \pmb {\Omega } \right) ^{-1} \pmb {\Delta }. \end{aligned}$$

Here, $\pmb {\Omega }$ represents a symmetric matrix with zeroes on the diagonal elements and partial correlation coefficients on the off-diagonal elements, and $\pmb {\Delta }$ represents a diagonal scaling matrix that controls the variances (and is a function of $\pmb {\Omega }$, as explained below in Eq. (10)). The $\pmb {\Omega }$ matrix can be used as a weight matrix to draw a network, in which nonzero elements of $\pmb {\Omega }$ are represented by an edge in the network representation. While $\pmb {\Omega }$ and $\pmb {P}$ can be directly transformed into one-another in principle, we have neither in practice; we merely have the estimate $\pmb {R}$. This estimate naturally contains noise due to sampling variation, but may also contain noise due to heterogeneity across samples (Becker 1992; 1995):

$$\begin{aligned} \pmb {R} = \pmb {P} + \text {sampling error} + \text {heterogeneity}. \end{aligned}$$

When analyzing only one dataset, heterogeneity across study domains cannot be taken into account. The extend of sampling error, however, can adequately be estimated through various methods. A classical method of controlling for sampling error is by obtaining maximum likelihood estimates of $\pmb {\Omega }$ as well as standard errors around each of these estimates, which can subsequently be used to assess the significance of parameter values. The exact same procedure could also be used to test confirmatory fit of a pre-defined structure for $\pmb {\Omega }$ in which some parameters are constrained to zero based on, for example, a cross-validation training dataset (Kan et al. 2019; Kan et al. 2020). Several fit indices could then be obtained for assessing the fit of the model (Howard, 2013). It has been noted, however, that the methods and software typically used to estimate GGMs lack this classical level of inference (Williams and Rast 2018), relying instead on regularization techniques and data driven re-sampling methods (Epskamp et al. 2018). Epskamp et al. (2017), for example, do not report analytic derivatives of the model in Eq. (1) that are required for this level of inference. After introducing a general modeling framework in Sect. 2, in which all models discussed in this paper are embedded, we fully describe these analytic derivatives in Sect. 3, and present a less technical introduction to these methods in Supplement 1.

Extending the problem to multiple datasets, we introduce the *meta-analytic Gaussian network aggregation* (MAGNA) framework, which is derived from earlier work on multi-group structural equation modeling (SEM; Bollen and Stine 1993) and meta-analytic SEM (MASEM; Cheung 2015a; Cheung and Chan 2005). We discuss two variants of MAGNA: fixed-effects MAGNA (Sect. 4) and random-effects MAGNA (Sect. 5). In the fixed-effects MAGNA setting, we do not assume heterogeneity across study domains, and aim to estimate a single GGM using multi-dataset analysis, either by estimating a pooled correlation structure to use in GGM estimation, or by estimating a single GGM directly in a multi-dataset GGM model using equality constraints across datasets. In the later variant, we can also place partial equality constraints, allowing for some parameters to be equal across groups while others vary across groups. In the random-effects MAGNA setting, we assume heterogeneity across study domains, and aim to estimate a GGM structure while taking this heterogeneity into account. To do this, we need a prior estimate of the sampling error among sample correlation coefficients, which can be obtained using the methods discussed in Sects. 2, 3, and 4.

Following the introduction of the MAGNA framework, Sect. 6 reports simulation results on the performance of fixed-effects and random-effects MAGNA analysis from datasets with and without heterogeneity. This is the first simulation study that incorporates cross-study heterogeneity in GGM estimation procedures. We will discuss and implications for the performance of aggregating over studies while not controlling for cross-study heterogeneity. Finally, Sect. 7 discusses two empirical applications of PTSD symptom networks, and Supplement 4 discusses another empirical example of depression, anxiety and stress symptoms.

All methods have been implemented in the open-source R package *psychonetrics* (Epskamp 2020b).^3^ A tutorial on how all analyses can be performed using *psychonetrics* can be found in Supplement 2, and more information on estimating models with missing data can be found in Supplement 3. The analytical framework from Sect. 2 can further be used for other models than the GGM; in Supplement 5 we detail how this framework can be used for another common network model—the Ising Model for dichotomous data (Epskamp et al. 2018; Ising 1925; Marsman et al. 2018). This Supplement explains how the Ising model can be estimated from summary statistics as well as how it can be extended to multi-dataset analysis—both types of analyses not previously used in the literature on psychological network analysis.

## Notation

Throughout this paper and the supplementary materials, we will use Roman letters to denote variables that can be observed (such as data and sample size), and Greek letters to denote parameters that are not observed. Normal faced letters will be used to denote scalars, bold-faced lower-case letters to denote vectors, and bold-faced upper-case letters to denote matrices. In line with earlier work on psychometric network models (Epskamp, 2020a; Epskamp et al. 2018), we used capitalized subscripts to denote that a variable is random with respect to that population. For example, $\pmb {y}_C$ denotes that the response vector $\pmb {y}$ is random with respect to case *C*, and $\pmb {y}_c$ denotes the observed response vector from a fixed case *c*. In addition, we will use some common vectors and matrices: $\pmb {I}$ represents an identity matrix, $\pmb {O}$ a matrix of zeroes, and $\pmb {0}$ a vector of zeroes. The symbol $\otimes$ will be used to denote the Kronecker product. We will also use some matrix functions: $\mathrm {vec}(\ldots )$ will represent the column-stacked vectorization operator, $\mathrm {vech}(\ldots )$ the column-stacked half-vectorization operator (lower triangular elements including the diagonal), $\mathrm {vechs}(\ldots )$ the strict column-stacked half-vectorization operator (lower triangular elements omitting the diagonal), and $\mathrm {diag}(\ldots )$ will return only the diagonal elements of a matrix.

## A General Framework for Structured Multivariate Models

In this section, we introduce a general framework for maximum likelihood estimation of structured multivariate models, such as all the models discussed in the present paper. This framework is based on commonly used frameworks for estimating multivariate models (Magnus and Neudecker 1999; Neudecker and Satorra 1991). We introduce this framework here, however, as to keep the paper self-contained. All models introduced after this section follow the framework introduced here. In fact, the random-effects MAGNA framework uses this framework twice in different ways. First, to estimate the sampling error around sample correlation coefficients. Second, to estimated the remaining parameters. We further introduce this framework first without assuming an underlying distribution, as this allows the framework to be used flexibly for standardized (e.g., estimating a GGM from a correlation matrix) and unstandardized (e.g., modeling variance around multiple correlation coefficients) Gaussian distributions. Supplement 3 continues the discussion of this chapter and shows how the framework can be expanded to handle missing data and case-specific distributions. Finally, this specification also allows for non-Gaussian distributions to be used, as is further described in Supplement 5, which uses the framework for dichotomous Ising models instead.

**Fit function.** Let $\pmb {\mathcal {D}}$ represent all available data and let $\mathcal {L}$ represent the log-likelihood of the data, which we assume to follow a multivariate distribution that is characterized by of *distribution parameters* $\pmb {\phi }$ (e.g., all population correlation coefficients). We will model the data using a set of *model parameters* $\pmb {\theta }$ (e.g., all possible edges in a GGM network), which are subsequently modeled with a set of *free parameters* $\pmb {\psi }$ (e.g., all ‘included’ nonzero edges in a GGM network). As such, $\mathcal {L}$ is a function of $\pmb {\phi }$, which is a function of $\pmb {\theta }$, which, finally, is a function of $\pmb {\psi }$:

$$\begin{aligned} \mathcal {L}\left( \pmb {\phi }\left( \pmb {\theta }\left( \pmb {\psi } \right) \right) ; \pmb {\mathcal {D}} \right) . \end{aligned}$$

We will drop or reduce bracket notation for functions whenever non-ambiguous. For example, the above can also be written as $\mathcal {L}\left( \pmb {\psi } ; \pmb {\mathcal {D}} \right)$ (as the likelihood is ultimately a function of *free parameters* in $\pmb {\psi }$ only) or simply $\mathcal {L}$. Rather than using the log-likelihood itself, we will use a fit function that is proportional to $-2 / n$ times the log-likelihood, with *n* representing the total sample size:^4^

2 $$\begin{aligned} F \propto - \frac{2}{n} \mathcal {L}. \end{aligned}$$

**Derivatives of the gradient.** In maximum likelihood estimation (ML), we find parameters by minimizing *F*:

$$\begin{aligned} \hat{\pmb {\psi }} = \min _{\pmb {\psi }} \left[ F( \pmb {\psi }; \pmb {\mathcal {D}} ) \right] \end{aligned}$$

which we can do by finding the set of parameters for which the gradient—the transpose of the first-order derivative (Jacobian)—equals $\pmb {0}$:

3 $$\begin{aligned} \pmb {\nabla } F\left( \hat{\pmb {\psi }} \right) = \left( \frac{\partial F\left( \hat{\pmb {\psi }} \right) }{\partial \pmb {\psi }} \right) ^{\top } = \pmb {0}. \end{aligned}$$

Numerous algorithms exist for solving a gradient to be $\pmb {0}$.^5^ This Jacobian matrix can be found using a chain-rule (Neudecker and Satorra 1991):

4 $$\begin{aligned} \frac{\partial F}{\partial \pmb {\psi }} =&\text {Full Jacobian} \nonumber \\&\left( \frac{\partial F}{\partial \pmb {\phi }} \right) \times&\text {Distribution Jacobian} \nonumber \\&\left( \frac{\partial \pmb {\phi }}{\partial \pmb {\theta }}\right) \times&\text {Model Jacobian} \nonumber \\&\left( \frac{\partial \pmb {\theta }}{\partial \pmb {\psi }}\right)&\text {Manual Jacobian}. \end{aligned}$$

Three elements are needed to obtain the Jacobian: the *distribution Jacobian* (e.g. the derivative of the normal likelihood to the means, variances and covariances), the *model Jacobian* (e.g., the derivative of correlations to network edge weights), and the *manual Jacobian* (e.g., the derivative of all possible network edge weights to unique nonzero edge weights). The *manual Jacobian* allows for constraining parameters (e.g., to zero) and for specifying equality constraints, and will usually be a sparse matrix consisting only of ones (parameters that are estimated) and zeroes (parameters that are fixed to their starting values, usually zero). The distribution and *model Jacobian*s need to be defined. Note that in the special case where $\pmb {\phi } = \pmb {\theta } = \pmb {\psi }$ (e.g., when all correlations are directly modeled), the *model Jacobian* and *manual Jacobian* both become an identity matrix.

**Fisher information and standard errors.** The full Jacobian above is sufficient for relatively fast parameter estimation. However, to obtain standard errors of the estimated parameters we also require second-order derivatives. The Hessian denotes the second-order derivative of the fit function (Jacobian of the gradient):

$$\begin{aligned} \pmb {H} = \frac{\partial \pmb {\nabla } F }{\partial \pmb {\psi }}. \end{aligned}$$

The expected value ($\mathcal {E}$) of the Hessian can be used to obtain the unit Fisher information matrix:

$$\begin{aligned} \pmb {\mathcal {I}} = \frac{1}{2} \mathcal {E}\left( \pmb {H} \right) , \end{aligned}$$

While the full Hessian is hard to compute, a convenient chain-rule exists for the Fisher information of the maximum likelihood estimate $\hat{\pmb {\psi }}$, making use of Eq. (3) such that the gradient equals zero (Magnus and Neudecker 1999):

5 $$\begin{aligned} \pmb {\mathcal {I}}\left( \hat{\pmb {\psi }}\right) = \frac{1}{2} \left( \frac{\partial \pmb {\theta }}{\partial \pmb {\psi }}\right) ^{\top } \left( \frac{\partial \pmb {\phi }}{\partial \pmb {\theta }}\right) ^{\top } \left( \mathcal {E}\left[ \frac{\partial \pmb {\nabla } F\left( \hat{\pmb {\psi }}\right) }{\partial \pmb {\phi }} \right] \right) \left( \frac{\partial \pmb {\phi }}{\partial \pmb {\theta }}\right) \left( \frac{\partial \pmb {\theta }}{\partial \pmb {\psi }}\right) . \end{aligned}$$

As such, only one more matrix is needed: a second-order derivative of the fit function to the *distribution parameters* only, which we will term the *Distribution Hessian*. The Fisher information can subsequently be used to obtain an estimate of the parameter variance–covariance matrix of the maximum likelihood estimate $\hat{\pmb {\psi }}$:

6 $$\begin{aligned} \pmb {\mathcal {V}}\left( \hat{\pmb {\psi }} \right) = \frac{1}{n} \pmb {\mathcal {I}}\left( \hat{\pmb {\psi }} \right) ^{-1} \approx \mathrm {var}\left( \hat{\pmb {\psi }} \right) . \end{aligned}$$

The square root of the diagonal of this matrix can be used to estimate the standard error of each free parameter. Of note, the above expression should be read as the variance of the ML *estimator* of $\pmb {\psi }$, not the variance of the ML *estimate* $\hat{\pmb {\psi }}$. The ML estimate $\hat{\pmb {\psi }}$ is deterministic for a given dataset, and thus fixed without variance. However, if the study is repeated in the exact same setting in the same population, sampling variation will lead $\hat{\pmb {\psi }}$ to vary across these potential samples. These potential samples do not necessarily equal the multiple datasets discussed in this paper, as there may be differences in the populations studied in different datasets.

**Summary** To summarize, this section describes a general modeling framework that only needs the implementation of the *distribution Jacobian* and Hessian for each specific distribution, the implementation of the *model Jacobian* for each specific model, and the specification of the *manual Jacobian* for each specification of the model. This framework is implemented in the *psychonetrics* R package (Epskamp 2020b), which now contains two distributions: the Gaussian distribution introduced further below, and the Ising distribution introduced in Supplement 5. The package furthermore includes several modeling frameworks based on these distributions (mostly network models and latent variable models; Epskamp 2020a). This paper will focus only on the Gaussian distribution coupled with the Gaussian graphical model.

## Single Dataset ML Estimation

In this section, we will discuss ML estimation in a single dataset. We discuss the single dataset case first, as the methods for multi-dataset and meta-analytic analyses discussed further in this paper naturally follow from the methods for single group analysis. An example of how the methods below can be used to estimate and perform inference on a GGM structure based on an observed correlation matrix can be seen in Supplement 1. Let $$\pmb {y}^{\top }_c = \begin{bmatrix} y_{[c,1]}&y_{[c,2]}&\ldots&y_{[c,p]} \end{bmatrix}$$ represent the response vector of case *c* on a set of *p* items, and let $\pmb {Y}$ represent the data matrix that contains these responses on its rows:

$$\begin{aligned} \pmb {Y} = \begin{bmatrix} \pmb {y}^{\top }_1 \\ \pmb {y}^{\top }_2 \\ \vdots \\ \pmb {y}^{\top }_n \end{bmatrix} = \begin{bmatrix} y_{[1,1]} &{}\quad y_{[1,2]} &{}\quad \ldots &{}\quad y_{[1,p]} \\ y_{[2,1]} &{}\quad y_{[2,2]} &{}\quad \ldots &{}\quad y_{[2,p]} \\ \vdots &{}\vdots &{}\vdots &{}\vdots \\ y_{[n,1]} &{} \quad y_{[n,2]} &{}\quad \ldots &{}\quad y_{[n,p]} \end{bmatrix}. \end{aligned}$$

As we only consider one dataset, $\pmb {\mathcal {D}} = \pmb {Y}$. We will assume that $\pmb {Y}$ contains no missing data,^6^ and that $\pmb {Y}$ is standardized such that the sample mean of each variable is 0 and the standard deviation^7^ of each variable is 1. We will first discuss the fit function and derivatives for models that utilize the standardized Gaussian distribution. Next, we discuss estimating GGMs with potentially constrained structures. Finally, we also discuss how potentially constrained marginal correlation models can be estimated in this framework.

### The Standardized Gaussian Distribution

Let $\pmb {R}$ denote the sample correlation matrix of $\pmb {Y}$, obtained (if the data are standardized) with:

$$\begin{aligned} \pmb {R} = \frac{1}{n} \pmb {Y}^{\top } \pmb {Y}. \end{aligned}$$

As data are assumed standardized, we will assume that $\pmb {y}_{C}$ follows a multivariate standard normal distribution:

$$\begin{aligned} \pmb {y}_{C} \sim N(\pmb {0}, \pmb {P}), \end{aligned}$$

in which $\pmb {P}$ represents the population correlation matrix. In the case of standardized data, the only *distribution parameters* of interest are the correlation coefficients:

$$\begin{aligned} \pmb {\phi } = \pmb {\rho } = \mathrm {vechs}\left( \pmb {P} \right) . \end{aligned}$$

As a result, the fit function becomes:

7 $$\begin{aligned} F = \mathrm {trace}\left( \pmb {R} \pmb {K} \right) - \ln |\pmb {K}|, \end{aligned}$$

in which $\pmb {K} = \pmb {P}^{-1}$. Important to note here is that the fit function is only a function of the sample correlation matrix $\pmb {R}$ and no longer of raw data $\pmb {Y}$—the sample correlations are sufficient statistics for the standardized Gaussian distribution. The *distribution Jacobian* becomes:

8 $$\begin{aligned} \frac{\partial F}{\partial \pmb {\rho }} = - \mathrm {vec}\left( \pmb {K} \left( \pmb {R} - \pmb {P} \right) \pmb {K} \right) ^{\top } \pmb {D}_{*}, \end{aligned}$$

in which $\pmb {D}_{*}$ represents a strict duplication matrix as further discussed in the appendix. Finally, the *distribution Hessian* becomes:

9 $$\begin{aligned} \mathcal {E}\left[ \frac{\partial \pmb {\nabla } F }{\partial \pmb {\rho }} \right] = \pmb {D}_{*}^{\top } \left( \pmb {K} \otimes \pmb {K}\right) \pmb {D}_{*}. \end{aligned}$$

### The Gaussian Graphical Model

Equation (1) characterizes the GGM as a function of a symmetric matrix $\pmb {\Omega }$ with zeroes on the diagonal and partial correlation coefficients on the off-diagonal, and a diagonal scaling matrix $\pmb {\Delta }$. In the special case of modeling a correlation matrix, the $\pmb {\Delta }$ is a function of $\pmb {\Omega }$ such that all diagonal elements of $\pmb {P}$ equal 1:

10 $$\begin{aligned} \pmb {\Delta } = \mathrm {vec2diag}\left( \mathrm {diag}\left( \left( \pmb {I} - \pmb {\Omega } \right) ^{-1} \right) \right) ^{-\frac{1}{2}}, \end{aligned}$$

in which $\mathrm {vec2diag}(\ldots )$ takes a vector and returns a diagonal matrix with elements of the vector on the diagonal, and $\mathrm {diag}(\ldots )$ takes the diagonal of a matrix and returns a vector. As such, the only parameters in the model are $\pmb {\theta } = \pmb {\omega } = \mathrm {vechs}\left( \pmb {\Omega }\right)$. The *model Jacobian* can be derived to take the following form:

11 $$\begin{aligned}&\frac{\partial \pmb {\phi }}{\partial \pmb {\theta }} = \frac{\partial \pmb {\phi }}{\partial \pmb {\omega }} \nonumber \\&= \pmb {L}^{*} \left[ \left( \pmb {\Delta } \pmb {\Omega }^{*} \otimes \pmb {\Delta } \pmb {\Omega }^{*} \right) - 0.5 \left( \left( \pmb {\Delta }\pmb {\Omega }^{*} \otimes \pmb {I} \right) + \left( \pmb {I} \otimes \pmb {\Delta }\pmb {\Omega }^{*} \right) \right) \pmb {A} \mathrm {dmat}\left( \pmb {\Omega }^{*} \right) ^{-\frac{3}{2}} \pmb {A}^{\top }\left( \pmb {\Omega }^{*} \otimes \pmb {\Omega }^{*} \right) \right] \pmb {D}^{*} \end{aligned}$$

in which $\pmb {\Omega }^{*} = \left( \pmb {I} - \pmb {\Omega }\right) ^{-1}$. The $\mathrm {dmat}(\ldots )$ function returns a matrix that only includes the diagonal of the input (all other elements set to zero), and the power $-\frac{3}{2}$ is only taken for diagonal elements of the diagonal matrix. The matrices $\pmb {L}^{*}$ and $\pmb {A}$ are further explained in the appendix.

With Eq. (1) at hand, all elements required for estimating GGM structures with possible (equality) constraints among the parameters are there for both single-dataset and multiple-dataset models—explained further below in Sect. 4.2.2. To estimate the GGM parameters, we can numerically solve Eq. (3) using any good optimization routine, which equates to finding the set of parameters that maximises the likelihood function. To do this, we need to use Eq. (4), which expresses the gradient, and plug in the correct matrices: Eq. (8) for the *distribution Jacobian* and Eq. (11) for the *model Jacobian*. The *manual Jacobian* can finally be specified to encode which parameters are constrained in the model. This matrix will have a row for each potential edge in the network (each unique element in $\pmb {\Omega }$), and a column for each parameter that is free to vary. The matrix only contains ones and zeroes, with a one indicating that an element of $\pmb {\Omega }$ is represented by a free parameter in $\pmb {\psi }$. A diagonal matrix represents a saturated model, a diagonal matrix with columns cut out represents a model in which certain edges are fixed to zero, and a matrix in which multiple elements in a column are 1 represent a model with equality constraints.

For example, consider a hypothetical model for three variables, such that there are three distribution parameters: $$\pmb {\theta }^{\top } = \begin{bmatrix}\omega _{21}&\omega _{31}&\omega _{32} \end{bmatrix}$$. The following manual matrix specifications can be used to encode different constrained models for these parameters:The first specification will lead to a saturated model in which all three potential network edges are included ($\psi _1 = \omega _{21}, \psi _2 = \omega _{31}, \psi _3 = \omega _{32}$), the second specification will lead to a constrained model, in which only edges 1 – 2 ($\psi _1 = \omega _{21}$) and 2 – 3 ($\psi _2 = \omega _{32}$) are included, and the last specification will lead to a further constrained model in which these two edges are also constrained to be equal ($\psi = \omega _{21} = \omega _{32}$). After estimating a model, Eq. (5) can be used to compute the Fisher information matrix, which can be used in Eq. (6) to estimate standard errors of the parameters. We can plug in the same *manual Jacobian* and *model Jacobian* as for the gradient, in addition to Eq. (9) for the *distribution Hessian*. With standard errors of the parameters, we could assess which edges are not significantly different from zero at a given $\alpha$ level and re-estimate parameters of other edges while keeping the edge-weights from non-significant edges constrained to zero—a process we term *pruning*. Supplementary 1 shows a non-technical description of how to do this in a model with three variables, and Supplementary 2 shows a tutorial on how to do this in R using the *psychonetrics* package.

### Estimating Correlations

The expressions above can also be used to estimate correlation coefficients rather than GGM structures (partial correlation coefficients). For this, we only need to change the *model Jacobian* in the gradient and Fisher information expressions. If we do not impose any structure on the correlation coefficients (estimating a saturated model), we can see that then $\pmb {\phi } = \pmb {\theta } = \pmb {\psi } = \pmb {\rho }$, and therefore both the *model Jacobian* and the *manual Jacobian* equal an identity matrix. To this end, the transpose of Eq. (8) directly equals the gradient, which is solved for $\pmb {0}$ when $\pmb {P} = \pmb {R}$, proving that $\pmb {R}$ is a ML estimate for $\pmb {P}$. Equation (9) can then be used directly to form the parameter variance-covariance matrix $\pmb {\mathcal {V}}$, which can be used to obtain standard error estimates for the estimated correlations by taking the square root of diagonal elements. This is important for the discussion in this paper, as the multi-dataset MAGNA methods introduced below rely crucially on the $\pmb {\mathcal {V}}$ matrix for marginal correlation matrices. In fixed-effects MAGNA, we will use these expressions to estimate a pooled correlation matrix to estimate a GGM from, and in random-effects MAGNA, we will use these expressions to estimate the sampling variation among the correlational structure. Of note, constrained correlation models, such as fixing certain correlations to zero or imposing equality constraints between multiple correlations, can easily be estimated using this framework as well by changing the manual matrix.

## Multiple Dataset ML Estimation: Fixed-Effects MAGNA

When analyzing multiple datasets, we can form a set of parameters for each dataset and place equality constraints across datasets to estimate a (partly) identical model. This approach is common in the SEM literature, where multi-group analyses are frequently used to assess measurement invariance and heterogeneity across groups (Meredith 1993). We use multi-dataset analysis in the remainder of the paper in several different ways: to obtain a pooled correlation structure and weight matrix in Sect. 4.2.1, to estimate a (partly) identical GGM structure across multiple groups in Sect. 4.2.2, and to estimate sampling variation across different datasets in Sect. 5.2.2. To extend our analyses to accommodate multiple datasets, we may note that the framework presented in Sect. 2 does not necessarily require that only one dataset is present. This framework merely requires a likelihood function (e.g., the total likelihood over all datasets), a set of *distribution parameters* (e.g., the sample correlations from all datasets), a set of *model parameters* (e.g., edge weights for all datasets), and a set of free parameters (e.g., a single set of identical edge weights across groups). As such, this framework allows for the modelling of multiple datasets as well as single datasets. In addition, it turns out that this can be obtained with minimal adjustments to the required Jacobian and Hessian blocks; mostly the exact same structures as used in single-dataset estimation can be used in multiple-dataset estimation. Below, we first discuss this in more detail, before turning to the specific cases of estimating pooled correlations and GGMs.

### Modeling Multiple Matasets

Suppose we have not one but *k* datasets. We can then use subscript $i \in 1, 2, \ldots , k$ to distinguish between datasets. The full data then becomes a set of datasets:

$$\begin{aligned} \pmb {\mathcal {D}} = \left\{ \pmb {Y}_1, \pmb {Y}_2, \ldots , \pmb {Y}_{k} \right\} . \end{aligned}$$

Let $F_i$ indicate the likelihood function of dataset *i* with a sample size of $n_i$. We can then form a fit function for each dataset separately, $F_i$ (taking the form of, for example, Eq. (7)). Then, assuming independence between datasets, we may form the fit function of Eq. (2) of the full data as the weighted sum of fit functions over all datasets:

12 $$\begin{aligned} F = \sum _{i=1}^{k} \frac{n_i}{n} F_i. \end{aligned}$$

Each of these fit functions can have its own set of *distribution parameters* $\pmb {\phi }_i$ and *model parameters* $\pmb {\theta }_i$, such that:^8^

$$\begin{aligned} \pmb {\phi } = \begin{bmatrix} \pmb {\phi }_1 \\ \pmb {\phi }_2 \\ \vdots \\ \pmb {\phi }_{k} \end{bmatrix}, \pmb {\theta } = \begin{bmatrix} \pmb {\theta }_1 \\ \pmb {\theta }_2 \\ \vdots \\ \pmb {\theta }_{k} \end{bmatrix}. \end{aligned}$$

The *distribution Jacobian*, *model Jacobian*, and *distribution Hessian* each then become a block matrices:

13 $$\begin{aligned} \frac{\partial F}{\partial \pmb {\phi }}&= \begin{bmatrix} \frac{n_1}{n} \left( \frac{\partial F_1}{\partial \pmb {\phi }_1} \right)&\frac{n_2}{n} \left( \frac{\partial F_2}{\partial \pmb {\phi }_2} \right)&\ldots&\frac{n_{k}}{n} \left( \frac{\partial F_{k}}{\partial \pmb {\phi }_{k}} \right) \end{bmatrix} \end{aligned}$$

14 $$\begin{aligned} \frac{\partial \pmb {\phi }}{\partial \pmb {\theta }}&= \begin{bmatrix} \left( \frac{\partial \pmb {\phi }_1}{\partial \pmb {\theta }_1}\right) &{} \pmb {O} \ldots &{} \pmb {O} \\ \pmb {O} &{}\left( \frac{\partial \pmb {\phi }_2}{\partial \pmb {\theta }_2}\right) &{} \ldots &{} \pmb {O} \\ \vdots &{} \vdots &{} \ddots &{} \vdots \\ \pmb {O} &{} \pmb {O} &{} \ldots &{} \left( \frac{\partial \pmb {\phi }_{k}}{\partial \pmb {\theta }_{k}}\right) \end{bmatrix} \end{aligned}$$

15 $$\begin{aligned} \mathcal {E}\left[ \frac{\partial \pmb {\nabla } F }{\partial \pmb {\phi }} \right]&= \begin{bmatrix} \frac{n_1}{n} \left( \mathcal {E}\left[ \frac{\partial \pmb {\nabla } F_1 }{\partial \pmb {\phi }_1} \right] \right) &{} \pmb {O} &{} \ldots &{} \pmb {O} \\ \pmb {O} &{} \frac{n_2}{n} \left( \mathcal {E}\left[ \frac{\partial \pmb {\nabla } F_2 }{\partial \pmb {\phi }_2} \right] \right) &{} \ldots &{} \pmb {O} \\ \vdots &{} \vdots &{} \ddots &{} \vdots \\ \pmb {O} &{} \pmb {O} &{} \ldots &{} \frac{n_{k}}{n} \left( \mathcal {E}\left[ \frac{\partial \pmb {\nabla } F_{k} }{\partial \pmb {\phi }_{k}} \right] \right) \end{bmatrix}. \end{aligned}$$

As such, the *distribution Jacobian* and Hessian and the *model Jacobian* only need to be defined for each dataset separately. This greatly simplifies the multi-dataset estimation problem, as no new derivatives are required for the multi-dataset setting as for the single data-set setting. As a result, no new derivatives are required for, for example, multi-dataset correlation models (Sect. 4.2.1), GGM models (Sect. 4.2.2), and Ising models (Supplement 5). Finally, the *manual Jacobian* can be used to impose equality constraints over datasets. For example, suppose we wish to estimate a model in which each network edge is included but constrained to be equal across datasets, we could specify:16with $\pmb {I}$ indicating an identity matrix. If we then wish to fit a model in which some edges are constrained to zero over all groups as well, we only need to cut out columns of the *manual Jacobian* above.

Of note, any dataset can be split into multiple datasets as well. As such, estimating a single-dataset model on the full data or estimating a multiple dataset model on the data randomly split in two with parameters constrained to be equal across datasets should lead to the same estimates. This property can be used to implement full information maximum likelihood (FIML) estimation, which is typically used to handle missingness in the data. In FIML, each row of the dataset^9^ can be modeled as a dataset, and the methods above can be used to estimate the parameters of interest. Another application of FIML is that the fit function, gradient and Fisher information matrices can be computed per row individually. The estimator then no longer requires summary statistics, but rather the raw data itself. We make use of this variant of FIML in random-effects MAGNA in Sect. (5.2.2), where we use a different implied variance–covariance matrix per set of sample correlations based, in part, on the sample size used to determine that set of sample correlations. Supplement 3 explains FIML in more detail, and shows that the form of the *model Jacobian* stays the same.

### Fixed-Effects MAGNA

When modeling multiple standardized datasets, we may add a subscripts *i* to indicate that each dataset has its own population correlation and GGM structure:

$$\begin{aligned} \pmb {y}_{[C,i]}&\sim N(\pmb {0}, \pmb {P}_i) \\ \pmb {P}_i&= \pmb {\Delta }_i \left( \pmb {I} - \pmb {\Omega }_i \right) ^{-1} \pmb {\Delta }_i, \end{aligned}$$

in which $\pmb {\Delta }_i$ remains a function of $\pmb {\Omega }_i$ as per Eq. (10). We may be interested in estimating a single GGM structure $\pmb {\Omega }$ (including structured zeroes to indicate absent edges) to underlie the data, such that $\pmb {\Omega } = \pmb {\Omega }_1 = \pmb {\Omega }_2 = \cdots = \pmb {\Omega }_{k}$, implying also that the data follows a common population correlation structure $\pmb {P} = \pmb {P}_1 = \pmb {P}_2 = \ldots = \pmb {P}_{k}$. Such a model would correspond to a model in which deviations between the correlational structures of multiple datasets are solely due to sampling variation and not due to cross-study heterogeneity. Two methods can be used for this purpose: two-stage estimation and multi-dataset estimation. These are structurally near-identical, and both utilize the fitting procedure discussed in Sect. 4. For both methods, only the *manual Jacobian* needs to be specified and all other derivatives given in Sect. 3 can readily be used. We term these methods *fixed-effects meta-analytic Gaussian network aggregation* (fixed-effects MAGNA).^10^

When estimating a saturated GGM, both the two-stage approach and the multi-dataset approach will lead to the exact same estimates and standard errors, and usually the methods will lead to only minimal differences in constrained estimation (e.g., significance pruning). To this end, both methods can be used for fixed-effects MAGNA analysis. One benefit of the multi-dataset method is that it can also be used for partial pooling as well as to test for homogeneity across groups in invariance testing steps (Kan et al. 2019). We introduce an algorithm—partial pruning—for exploratively searching for such a partially constrained model below. The two-stage approach, on the other hand, is simpler and does not require software dedicated to multi-dataset GGM modeling, allows for sharing the pooled correlation matrix and weight matrix for easier reproduction of results, and allows for easier multi-dataset modeling where invariance is assessed across, for example, two pooled correlation matrices for datasets of two types (e.g., veterans and refugees). As such, the two-stage estimation procedure is simpler, while the multi-dataset estimation procedure is more sophisticated and can be expanded more.

#### Two-Stage Estimation

The first method is described as a two-stage approach in MASEM (Cheung and Chan 2005; 2009; Jak and Cheung 2020). This method uses the estimator from Sect. 2 twice: first in a multi-dataset setting to estimate a pooled correlation matrix together with its Fisher information matrix using maximum likelihood estimation, and second in a single-dataset setting estimating a pooled GGM using weighted least squares (WLS) estimation.

**Stage 1: Pooled correlations.** In the first stage of two-stage estimation, we estimate a single pooled population correlation matrix $\pmb {P}$ together with its Fisher information matrix. In this setup, the *distribution parameters* and *model parameters* both contain correlation coefficients for each dataset:

$$\begin{aligned} \pmb {\phi } = \pmb {\theta } = \begin{bmatrix} \pmb {\rho }_1 \\ \pmb {\rho }_2 \\ \vdots \\ \pmb {\rho }_{k} \end{bmatrix}, \end{aligned}$$

in which $\pmb {\rho }_i = \mathrm {vechs}\left( \pmb {P}_i\right)$. The free parameter set only contains the pooled correlations:

$$\begin{aligned} \pmb {\psi } = \pmb {\rho }. \end{aligned}$$

As such, the *model Jacobian* takes the form of an identity matrix, and the *manual Jacobian* takes the form of Eq. (16). The *distribution Jacobian* can be formed as in Eq. (13), with each element taking the form of Eq. (8) weighted by the proportional sample size of the corresponding dataset. These are all the elements needed for constructing the Jacobian (Eq. (4)), which can be used to construct the gradient (Eq. (3)) used in optimization procedures to estimate the parameters, which we term $\hat{\pmb {\rho }}$ below. For the Fisher information matrix $\pmb {\mathcal {I}}$ (Eq. (5)), the *distribution Hessian* can be constructed as in Eq. (15), with each element taking the form of Eq. (9) weighted by the sample size.

**Stage 2: Pooled GGM** In the second stage of estimation, we utilize WLS estimation in a single-dataset setting to estimate the (potentially constrained) GGM parameters. In WLS, we match a set of *distribution parameters* $\pmb {\phi }$ to a set of observed summary statistics in $\pmb {z}$. The fit function used in WLS is:

$$\begin{aligned} F = \left( \pmb {z} - \pmb {\phi }\right) ^{\top } \pmb {W} \left( \pmb {z} - \pmb {\phi }\right) , \end{aligned}$$

in which $\pmb {W}$ is a weight matrix that needs to be defined. If $\pmb {W} = \pmb {I}$, WLS is also called unweighted least squares (ULS), and if $\pmb {W}$ is diagonal, WLS is also called diagonally weighted least squares (DWLS). The *distribution Jacobian* is:

$$\begin{aligned} \frac{\partial F}{\partial \pmb {\phi }} = -2 \left( \pmb {z} - \pmb {\phi }\right) ^{\top } \pmb {W}, \end{aligned}$$

and the *distribution Hessian* is:

$$\begin{aligned} \mathcal {E}\left[ \frac{\partial \pmb {\nabla } F }{\partial \pmb {\phi }} \right] = 2 \pmb {W}. \end{aligned}$$

In the second stage of two-stage fixed-effects MAGNA, we use the estimates from the first stage as observed statistics ($\pmb {z} = \hat{\pmb {\rho }}$) and use the Fisher information matrix as weight matrix ($\pmb {W} = \pmb {\mathcal {I}}$).^11^ The remainder of the estimation procedure is exactly the same as described in Sect. 3.2 (i.e., the *model Jacobian* takes the form of Eq. (11)).

#### Multi-dataset Estimation

A second method to estimate a single pooled GGM is to perform a single multi-dataset analysis in which a GGM is estimated. This is done in exactly the same way as stage one of the two-stage analysis method described in Sect. 4.2.1, with the exception that the *model Jacobian* now takes the form of Eq. (11) for each dataset. Like in the two-stage estimation method, the *manual Jacobian* can be specified as in Eq. (16) to estimate a saturated (all edges included) GGM with equality constraints over all datasets. Alternatively, columns of the *manual Jacobian* can be cut out to constrain certain edges to zero over all datasets, or columns can be added for partial equality constraints (some parameters constrained equal across groups and some allowed to vary across groups).

For example, suppose we have two datasets measured on three variables, and wish to estimate a GGM. We thus observe 6 correlations (3 per dataset), and model in total 6 potential GGM edges (3 per dataset), leading to the model parameters $$\pmb {\theta }^{\top } = \begin{bmatrix} \omega _{21,1}&\omega _{31,1}&\omega _{32,1}&\omega _{21,2}&\omega _{31,2}&\omega _{32,2}\end{bmatrix}$$. Consider the following options for the *manual Jacobian*:The first *manual Jacobian* will specify three unique parameters (three columns), which represent the three edges in the GGM. Therefore, the first *manual Jacobian* will estimate one single pooled network structure over both datasets ($\psi _1 = \omega _{21,1} = \omega _{21,2}, \psi _2 = \omega _{31,1} = \omega _{31,2}, \psi _3 = \omega _{32,1} = \omega _{32,1}$). The second *manual Jacobian*, instead, will estimate a unique Jacobian for each dataset ($\psi _1 = \omega _{21,1} , \psi _2 = \omega _{31,1} , \psi _3 = \omega _{32,1}, \psi _4 = \omega _{21,2} , \psi _5 = \omega _{31,2} , \psi _6 = \omega _{32,2}$). Finally, the third *manual Jacobian* will estimate a partially pooled GGM structure in which the first edge (the edge between variables one and two) is uniquely estimated in both datasets and the remaining edges are constrained equal over both groups ($\psi _1 = \omega _{21,1} , \psi _2 = \omega _{31,1} = \omega _{31,2}, \psi _3 = \omega _{32,1} = \omega _{32,2}, \psi _4 = \omega _{21,2}$).

#### Partial Pruning

As described above, the multi-dataset estimation method allows also for partial equivalence across datasets: models in which some—but not all—edges are constrained to be equal across groups. As such, this method also opens the door to (partial) invariance testing across groups (Kan et al. 2019). If the fully constrained model across datasets is rejected, it may be of interest to explore which parameters can be freed across datasets such that an acceptable model can be found. We propose an exploratory algorithm for this purpose: *partial pruning*, which has been implemented in the partialprune function in the *psychonetrics* package. The algorithm is as follows:

1. Estimate a model with significance pruning for each dataset separately, following Sect. 3.2 (for more details, see Supplement 1 and Supplement 2.1).
2. Estimate a pooled multi-dataset model (Sect. 4.2.2) in which each edge that was included in at least one of the individual models is included, and all edges are constrained equal across groups.
3. In a stepwise fashion: compute modification indices for included edges in each dataset with equality constraints (this modification index indicates the expected improvement in fit if the edge weight is freely estimated in that particular dataset) and sum these for all possible parameters, such that a single index is obtained for each edge that is included and is currently constrained to be equal across datasets. Free this parameter across the datasets if this improves BIC, and repeat this process until BIC can no longer be improved.
4. Remove all edge weights across all datasets that are not significant at $\alpha = 0.05$ and estimated the final model.

While highly exploratory, the reliance on optimizing BIC coupled with the last pruning step ensures that the algorithm remains conservative. The BIC, in particular, has been shown to perform well in choosing between competing GGMs (Foygel and Drton 2010), and similar stepwise BIC optimization strategies have been shown to perform well in estimating a GGM structure while not overfitting the data (Isvoranu and Epskamp 2021). Nonetheless, we recommend caution when interpreting results from this algorithm and to treat these as exploratory findings. The algorithm has been independently validated by Haslbeck (2020), who shows that the algorithm is conservative and performs comparably to other methods for detecting differences between datasets.

## Multiple Dataset ML Estimation: Random-Effects MAGNA

When fixed-effects MAGNA is used to estimate a pooled GGM, it is assumed that the true correlational structure (variances are ignored and may differ) is identical across all datasets. This may not be plausible or warranted. Consider for example network analyses on PTSD symptoms. We may expect large heterogeneity across samples used to study PTSD symptoms. For examples, in Sect. 7.1 we study four datasets supplied by Fried et al. (2018) which span multiple countries and investigate patients with very different backgrounds and traumas (e.g., soldiers and refugees). Previous research showed that it should not be expected that these samples come from populations with the exact same network model (Forbes et al. 2021; Fried et al. 2018; Williams et al. 2020). If many correlation matrices are to be aggregated to estimate a pooled GGM structure, as would be the purpose in meta-analytic research, a method that takes heterogeneity into account is needed. To this end, the current section introduces *random-effects meta-analytic Gaussian network aggregation* (random-effects MAGNA), which takes into account that samples may differ more from one-another than can be expected due to sampling variation alone. In addition, while the fixed-effects model provides a conditional inference conditioning on the studies included in the meta-analysis, the random-effects model instead gives an unconditional inference beyond the studies included in the meta-analysis if it can be assumed that the studies are a representative sample from the pool of studies that could have been performed (Egger et al. 1997; Hedges and Vevea 1998).

### Modeling Sample Correlations

Random-effects MAGNA is based on one-stage MASEM (Jak and Cheung 2020), and takes the form of a multi-level model in which a random effect is placed on the *correlational structure*. As such, random-effects MAGNA is not a multi-level GGM estimation tool—only one common GGM is estimated—but it does allow for taking heterogeneity into account by modeling the variance of correlations across studies. To perform random-effects MAGNA, first a dataset is formed in which each row represents a set of observed correlations for a study:

$$\begin{aligned} \pmb {\mathcal {D}} = \begin{bmatrix} \pmb {r}_1^{\top } \\ \pmb {r}_2^{\top } \\ \vdots \\ \pmb {r}_{k}^{\top } \\ \end{bmatrix}, \end{aligned}$$

in which $\pmb {r}_i = \mathrm {vechs}\left( \pmb {R}_i \right)$ (the sample correlation matrix of study *i*). When a correlation is not included in a particular study, the corresponding element can be encoded as missing (e.g., NA in *R*) and FIML can be used (see Supplement 3). This marks a strong benefit of random-effects MAGNA over, for example, performing a meta-analysis for each partial correlation coefficient separately: not all data-sets need to contain all variables used in the model. Crucially, while in fixed-effects MAGNA we were only concerned with a fixed set of datasets, in random-effects MAGNA we treat the dataset itself as random and model this distribution explicitly with a multivariate normal distribution with a mean-vector $\pmb {\mu }$ and variance–covariance matrix $\pmb {\Sigma }_i$:^12^ The subscript on $\pmb {\Sigma }_i$ indicates that FIML estimation is used and that the variance–covariance matrix $\pmb {\Sigma }_i$ may differ across datasets to take into account that some correlations are estimated more accurately (due to larger sample sizes) than others. We could also add a subscript *i* on the expected correlation structure $\pmb {\mu }$ to model missing variables. We discuss two variants of random-effects MAGNA estimation, one in which $\pmb {\Sigma }_i$ differs for each dataset, and one in which these are assumed identical over all datasets ($\pmb {\Sigma }_1 = \pmb {\Sigma }_2 = \ldots = \pmb {\Sigma }$). The dataset-specific *distribution parameters* therefore become:

$$\begin{aligned} \pmb {\phi }_i = \begin{bmatrix} \pmb {\mu } \\ \pmb {\sigma }_i \end{bmatrix}, \end{aligned}$$

in which $\pmb {\sigma }_i = \mathrm {vech}\left( \pmb {\Sigma }_i\right)$ (note that the diagonal is included). As such, random-effects MAGNA actually takes the form of a single-dataset problem (although multiple-dataset estimation is used for estimating the sampling variation below as well), and the framework from Sect. (2) can directly be used to estimate the free parameters.

Important to note is that while this framework works well for parameter estimation and significance of individual parameters, it works less well for model comparison. This is because the fit function no longer directly relates to the likelihood of the data, and as a consequence fit measures derived from the fit function are questionable. In addition, because different types of data are modeled in fixed-effects and random-effects MAGNA, these models are not nested and it is not appropriate to compare them. However, another possible fixed-effects model could be obtained by fixing $\pmb {\Sigma }^{(ran)} = \pmb {O}$ in random-effects MAGNA. A likelihood ratio statistic could be used to test whether the population variance component is zero. Since it is tested on the boundary (Self and Liang 1987), the test statistic must be adjusted. This test, however, is not popular in meta-analysis and multilevel models since the choice of model is usually based on theoretical rather than statistical reasons. In addition, due to the high model complexity of the random-effects MAGNA model, such a test may not lead to appropriate results and may be severely under-powered (e.g., the fixed-effects model may be preferred too often). To this end, we only use random-effects MAGNA for parameter estimation and inference on the parameter level, but not to, for example, compare fixed-effects to random-effects models. For assessing the appropriateness of random-effects MAGNA, the fixed-effects framework from Sect. 4.2.2 could be used to judge the fit of the constrained model without random effects.

### Random-Effects MAGNA

The mean structure can be specified to be the implied correlational structure from Eq. (1):

17 $$\begin{aligned} \pmb {\mu } = \pmb {\rho } = \mathrm {vechs} \left( \pmb {\Delta } \left( \pmb {I} - \pmb {\Omega } \right) ^{-1} \pmb {\Delta } \right) . \end{aligned}$$

The variance–covariance structure requires some more consideration. As $\pmb {r}_i$ is a set of random sample correlations, we should take into account that these vary across studies due to sampling variation in addition to potential heterogeneity. We can call the variance–covariance matrix due to sampling variation $\pmb {V}_i$. Next, additional variation will be due to random-effect variation of the correlational structure, which we can term $\pmb {\Sigma }^{(\mathrm {ran})}$. This leads to the following decomposition (e.g., Becker 1992):

18 $$\begin{aligned} \pmb {\Sigma }_i = \pmb {V}_i + \pmb {\Sigma }^{(\mathrm {ran})} \end{aligned}$$

The sampling variance–covariance matrix $\pmb {V}_i$ will always be present—as sample correlation coefficients naturally are not estimated without error—and should be in line with expected sampling error of the sample correlation coefficients. The random effects variance–covariance matrix $\pmb {\Sigma }^{(\mathrm {ran})}$ can differ, however, and can contain large or small elements depending on the level of (assumed) heterogeneity. Of note, in the fixed-effects approach taken above we implicitly assumed $\pmb {\Sigma }^{(\mathrm {ran})} = \pmb {O}$.

For estimating the random-effects MAGNA we will use a two-step approach, in which we first estimate $\pmb {V}_i$ separately, and subsequently treat this estimate as known in a second step in which the remaining model matrices are estimated. We make use of this two-step approach, because the structure of sampling variation can be well estimated before estimating other parameters in the model, and because it would otherwise not be possible to estimate both sampling variation and heterogeneity (co)variance parameters. Below, we will first outline how the random effects are modeled in Sect. 5.2.1. Next, we will discuss how $\pmb {V}_i$ can be estimated in Sect. 5.2.2. Finally, we discuss the required derivatives for parameter estimation in Sects. 5.2.3 and 5.2.3.

#### Model Setup

To ensure $\pmb {\Sigma }^{(\mathrm {ran})}$ is positive semi-definite, we will model this matrix using a Cholesky decomposition:

19 $$\begin{aligned} \pmb {\Sigma }^{(\mathrm {ran})} = \pmb {T} \pmb {T}^{\top }, \end{aligned}$$

in which $\pmb {T}$ is a lower triangular matrix with unique parameters $\pmb {\tau } = \mathrm {vech}\left( \pmb {T} \right)$ (not to be confused with $\tau ^2$, which is sometimes used to denote sampling variation in meta-analyses). As such, treating $\pmb {V}_i$ as known, the set of *model parameters* becomes:

$$\begin{aligned} \pmb {\theta } = \begin{bmatrix} \pmb {\omega } \\ \pmb {\tau } \end{bmatrix}. \end{aligned}$$

The set of free parameters, $\pmb {\psi }$, will contain all elements of $\pmb {\tau }$, which we estimate without constraints, and either all elements of $\pmb {\omega }$ or a subset of elements of $\pmb {\omega }$ indicating edge-weights that are nonzero.

#### Handling Sampling Variation

We can take two approaches in utilizing estimates for $\pmb {V}_i$ in estimating the random-effects MAGNA model (see e.g., Hafdahl 2008). First, we can form an estimate for each individual study $\widehat{\pmb {V}}_i$ and utilize FIML estimation in which the variance–covariance structure is specified differently *per study*:

20 $$\begin{aligned} \pmb {\Sigma }_i = \widehat{\pmb {V}}_i + \pmb {\Sigma }^{(\mathrm {ran})}. \end{aligned}$$

Second, we can form a single *averaged* estimate for $\pmb {V}_i$ that is the same for each study (dropping subscript *i*), $\widehat{\pmb {V}}_*$, which we can plug into (18) such that regular ML estimation can be used:

$$\begin{aligned} \pmb {\Sigma } = \widehat{\pmb {V}}_* + \pmb {\Sigma }^{(\mathrm {ran})}. \end{aligned}$$

The *averaged* approach implicitly assumes that sampling variation is the same across studies, and may not adequately take large differences in sample size into account. The *per study* approach, on the other hand, is computationally more challenging and may lead sooner to numeric optimization problems.

There are two ways in which the estimates $\widehat{\pmb {V}}_i$ and $\widehat{\pmb {V}}_*$ can be obtained. In *individual* estimation, we fit an unconstrained correlation model to each dataset separately as described in Sect. 3.3. An estimate for $\widehat{\pmb {V}}_i$ can then be directly obtained from the parameter variance–covariance matrix $\pmb {\mathcal {V}}$ described in Eq. (9). Following, an estimate for $\widehat{\pmb {V}}_*$ can be obtained by averaging these estimates:

$$\begin{aligned} \widehat{\pmb {V}}_* = \frac{1}{k} \sum _{i=1}^{k} \widehat{\pmb {V}}_i. \end{aligned}$$

If desired, a different averaging function can also be used, such as taking a weighted average in which the individual estimates are weighted with the sample size. In *pooled* estimation, we fit a multi-dataset pooled correlation model as described in stage 1 of two-stage fixed-effects MAGNA estimation (Sect. 4.2.1), and obtain an estimate for $\widehat{\pmb {V}}_*$ by multiplying the estimated parameter variance–covariance matrix (obtained by plugging the Fisher information matrix from Sect. 4.2.1 in Eq. (6)) with the number of datasets. Estimates for the dataset specific sampling variation matrices can then be obtained by weighing this estimate:

$$\begin{aligned} \widehat{\pmb {V}}_i = \frac{{\bar{n}}}{n_i} \widehat{\pmb {V}}_*, \end{aligned}$$

in which ${\bar{n}} = n / k$ is the average sample size.

In sum, when dealing with the sampling error matrices there are two different estimation procedures that can be used: *averaged* estimation in which the implied variance–covariance structure is the same over all datasets, and estimation *per study* in which the implied variance–covariance structure differs across datasets. There are also two different methods for constructing the sampling variation estimates: *individual* formation, in which the estimate is formed per study (and averaged for averaged estimation), and *pooled* estimation, in which the pooled model is used to estimate one sampling variation matrix (which can be weighted per study).

#### Derivatives of the Random-Effects MAGNA Model

With an estimate of $\pmb {V}$ at hand, we can now determine the required derivatives for estimating the parameters of interest (network parameters in $\pmb {\omega }$ and the Cholesky decomposition of random-effects variances and covariances in $\pmb {\tau }$). To do this, we can numerically solve Eq. (3), in which the gradient is formed as using the Jacobian elements described in Eq. (4). Subsequently, we can assess significance of parameters by forming the parameter variance–covariance matrix from Eq. (6), which uses the Fisher information formed in Eq. (5). In this section, we discuss the fit function and all required derivatives for this optimization problem.

First, we discuss the *averaged* estimation routine, in which a single estimate $\widehat{\pmb {V}}_*$ is used across all studies. Let $\bar{\pmb {r}}$ denotes the average sample correlation coefficient:

$$\begin{aligned} \bar{\pmb {r}} = \frac{1}{k} \sum _{i=1}^{k} \pmb {r}_i, \end{aligned}$$

and let $\pmb {S}$ represent the variance-covariance matrix of sample correlations:

$$\begin{aligned} \pmb {S} = \frac{1}{k} \sum _{i=1}^{k} (\pmb {r}_i - \bar{\pmb {r}} ) (\pmb {r}_i - \bar{\pmb {r}} )^{\top }, \end{aligned}$$

the fit function then becomes:

21 $$\begin{aligned} F = \mathrm {trace}\left( \pmb {S} \pmb {K} \right) + \left( \bar{\pmb {r}} - \pmb {\mu }\right) ^\top \pmb {K}\left( \bar{\pmb {r}} - \pmb {\mu }\right) - \ln |\pmb {K}|, \end{aligned}$$

in which $\pmb {K}$ now represents the inverse of $\pmb {\Sigma }$. The *distribution Jacobian* then becomes:

$$\begin{aligned} \frac{\partial F}{\partial \pmb {\phi }} = \begin{bmatrix} \left( \frac{\partial F}{\partial \pmb {\mu }} \right)&\left( \frac{\partial F}{\partial \pmb {\sigma }} \right) \end{bmatrix}, \end{aligned}$$

with the following blocks:

$$\begin{aligned} \left( \frac{\partial F}{\partial \pmb {\mu }} \right)&= - 2 \left( \bar{\pmb {r}} - \pmb {\mu } \right) ^{\top }\pmb {K} \\ \left( \frac{\partial F}{\partial \pmb {\sigma }} \right)&= - \mathrm {vec}\left( \pmb {K} \left( \pmb {S} + (\bar{\pmb {r}} - \pmb {\mu }) (\bar{\pmb {r}} - \pmb {\mu })^\top - \pmb {\Sigma } \right) \pmb {K} \right) ^{\top } \pmb {D}, \end{aligned}$$

in which $\pmb {D}$ is a *duplication matrix*, further discussed in the appendix. The *distribution Hessian* becomes:

$$\begin{aligned} \mathcal {E}\left[ \frac{\partial \pmb {\nabla } F }{\partial \pmb {\phi }} \right] = \begin{bmatrix} \pmb {H}_{\pmb {\mu }} &{} \pmb {O} \\ \pmb {O} &{} \pmb {H}_{\pmb {\sigma }}, \end{bmatrix} \end{aligned}$$

with the following blocks:

$$\begin{aligned} \pmb {H}_{\pmb {\mu }}&= 2 \pmb {K} \\ \pmb {H}_{\pmb {\sigma }}&= \pmb {D}^{\top } \left( \pmb {K} \otimes \pmb {K}\right) \pmb {D}. \end{aligned}$$

The *model Jacobian* takes the following form:

$$\begin{aligned} \frac{\partial \pmb {\phi }}{\partial \pmb {\theta }} = \begin{bmatrix} \left( \frac{\partial \pmb {\mu }}{\partial \pmb {\omega }} \right) &{} \pmb {O} \\ \pmb {O} &{} \left( \frac{\partial \pmb {\sigma }}{\partial \pmb {\tau }} \right) \end{bmatrix}, \end{aligned}$$

We may recognize that the mean structure from Eq. (17) takes the form of the strict half-vectorized GGM structure of Eq. (1). As such, the block $\partial \pmb {\mu } / \partial \pmb {\omega }$ takes the exact same form as in Eq. (11). The *model Jacobian* for the Cholesky decomposition becomes:

$$\begin{aligned} \frac{\partial \pmb {\sigma }}{\partial \pmb {\tau }} = \pmb {L} \left( \left( \pmb {I} \otimes \pmb {I} \right) + \pmb {C} \right) \left( \left( \pmb {T} \otimes \pmb {I} \right) \pmb {L}^{\top } \right) , \end{aligned}$$

in which $\pmb {C}$ is a commutation matrix as further discussed in the appendix.

For estimation *per study*, we use a separate estimate $\widehat{\pmb {V}}_i$ and utilize FIML estimation. Supplement 3 details FIML estimation in more detail, and shows that only study-specific variants of the fit function, *distribution Jacobian* and *distribution Hessian* are required. These can readily be obtained using the same derivatives described above by replacing $\bar{\pmb {r}}$ with $\pmb {r}_i$, $\pmb {S}$ with $\pmb {O}$, $\pmb {\mu }$ with $\pmb {\mu }_i$ (a subset of $\pmb {\mu }$ in the case there are missing variables in dataset *i*), and $\pmb {K}$ with $\pmb {K}_i$ (the inverse of $\pmb {\Sigma }_i$ as formed as per Eq. (20)). For example, making these changes makes the study-specific fit function from Eq. (21):

$$\begin{aligned} F_i = \left( \pmb {r}_i - \pmb {\mu }_i\right) ^\top \pmb {K}_i\left( \pmb {r}_i - \pmb {\mu }_i\right) - \ln |\pmb {K}_i|. \end{aligned}$$

The *distribution Jacobian* and *distribution Hessian* can similarly be formed in this manner and need not be discussed seperatly in more detail.

### Estimating the Random-Effects MAGNA Model

To summarize the above, in random-effects MAGNA we treat the observed sample correlation coefficients as the data. We model the expected value of these correlation matrices through a GGM, of which potentially some elements are constrained to zero (pruned model). The variance–covariance structure is modeled in two parts: a matrix of variation due to sampling-variation, and a matrix of variation due to heterogeneity. We make use of prior estimates of the sampling-variation matrix when estimating the heterogeneity matrix, which is modeled through the use of a Cholesky decomposition. The sampling-variation is formed either with *individual* estimation of an unconstrained correlation model for each individual study, or *pooled* estimation by estimating a single pooled correlation model across studies. Subsequently, the matrix can be used in estimation *per study*, evaluating the likelihood per study, or *averaged* estimation, in which a single estimate of the sampling variation matrix is used. This leads to four variants of random-effects MAGNA, which we assess in more detail below in simulation studies.

## Simulation Study

**Fig. 1:**
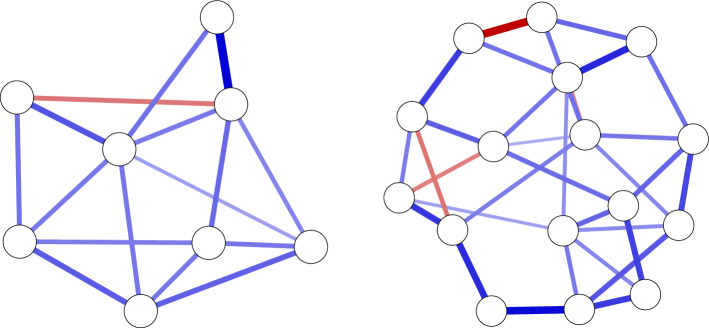
Example of true network structures used in simulation studies.

We performed a simulation study to assess the performance of fixed-effects and random-effects MAGNA. In each replication, we generated a true network structure using the *bootnet* package: 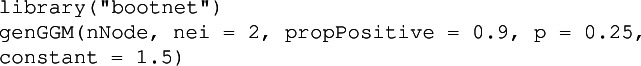 The structure is set up according to the Watts-Strogatz network model (Watts and Strogatz 1998), which starts the structure with nodes placed in a circle connected to their four nearest neighbors (the nei argument), and subsequently rewiring $25\%$ of the edges at random (the p argument). The algorithm of Yin and Li (2011) is used to weight the edges, with a constant of 1.5 and $90\%$ of the edges simulated to be positive (constant and propPositive arguments). We varied the number of nodes between 8 and 16; the procedure generated 8-node networks with 16 out of 28 potential edges ($57.1\%$) and 16-node networks with 32 out of 120 potential edges ($26.7\%$). This algorithm leads to an average absolute edge weight of 0.17. An example of networks generated with this procedure can be seen in Fig. 1. When simulating correlated random effects, the random effects variance–covariance matrix was generated using the rcorrmatrix function from the *clusterGeneration* package (Joe 2006; Qiu and Joe 2015): 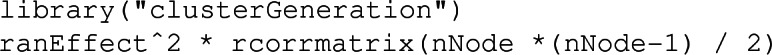 in which ranEffect indicates the random effect standard deviation for all correlations.

We assessed the performance of GGM estimation with significance pruning at $\alpha = 0.05$ using two variants of fixed-effects MAGNA (two-stage and multi-dataset) and four variants of random-effects MAGNA (varying sampling variance construction and estimation methods). We generated correlated random effects with random effect standard deviations varied between 0, 0.05, and 0.1 (higher values often lead to non-positive definite correlation matrices to be generated). We varied the number of datasets between 4, 8, 16, 32, and 64 with each dataset consisting of a sample size randomly chosen between $n_i = 250$ and $n_i = 1{,}000$. Each condition was replicated 100 times, leading to a total of 2 (number of nodes) $\times 5$ (number of datasets) $\times 3$ (random effect size) $\times 6$ (estimation method) $\times 100$ (replications) $= 18{,}000$ generated datasets. We assessed for each dataset the *absolute correlation* between estimated and true edge weights, the *sensitivity* (proportion of true edges detected in the estimated network, also termed “true positive rate”), the *specificity* (proportion of true zeroes detected in the estimated network, also termed “true negative rate”), and the average absolute bias in the standard error estimates of random effects.

**Fig. 2:**
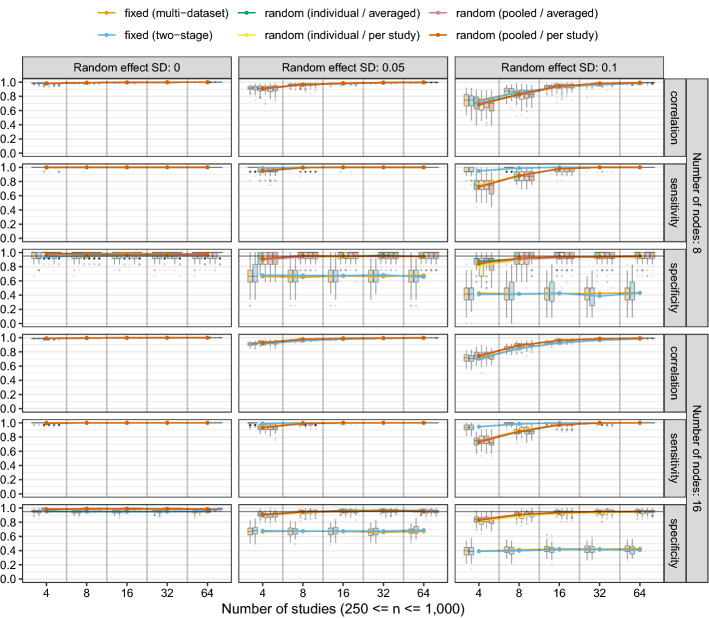
Results of the simulation study using significance pruning at $\alpha = 0.05$. Lines indicate means over all replications and boxplots indicate the spread across replications. Correlation indicates the correlation between absolute true and estimated edge weights in the pooled GGM, sensitivity—also termed the “true positive rate”—indicates the proportion of true edges included the estimated GGM, and specificity—also termed the “true negative rate”—indicates the proportion of true absent edges correctly not included in the estimated GGM.

**Fig. 3:**
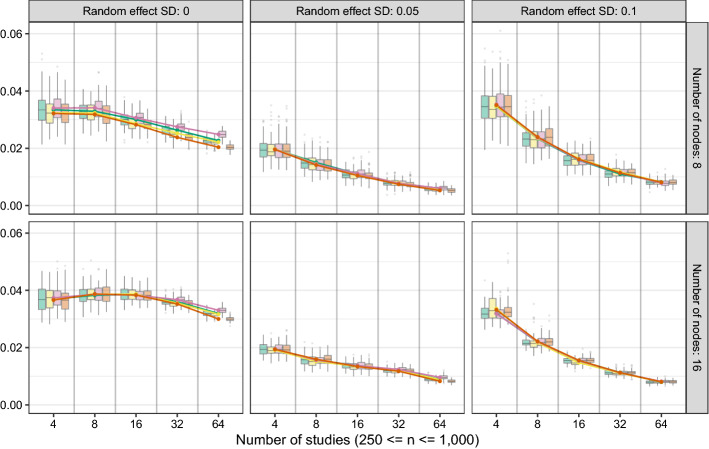
Average absolute deviations between estimated and true random effect standard deviations across all four random-effects MAGNA methods. The legend is the same as in Fig. 2.

Figure 2 shows the results for the correlation, sensitivity and specificity metrics, and Fig. 3 shows the results from random effect standard deviation bias assessment. The black horizontal line in the specificity panels of Fig. 2 highlights the expected specificity level of $1 - \alpha = 0.95$. A first thing to notice about the figures is that both fixed-effects MAGNA methods perform interchangeably, as do all four random-effects MAGNA methods. To this end, we will not discuss the methods specifically but rather limit discussion to fixed-effects MAGNA and random-effects MAGNA methods only. We will first discuss results from fixed-effects MAGNA, after which we turn evaluate random-effects MAGNA.

- **Fixed-effects MAGNA without cross-study heterogeneity** Investigating only the performance of the two fixed-effects MAGNA methods in estimating a pooled network structure in settings where the fixed-effects model is true, we can look at the “Random effect SD: 0” panels of Fig. 2. These show a remarkably strong performance of both fixed-effects MAGNA methods across the board: for any number of studies, sensitivity and edge-weight correlations are on average near 1, and specificity is on average exactly at the expected level of 0.95. To this end, fixed-effects MAGNA can be shown to perform well when the fixed-effects MAGNA model is true.
- **Fixed-effects MAGNA with cross-study heterogeneity** Investigating the other panels of Fig. 2 shows that the performance of fixed-effects MAGNA drops markedly with added cross-study heterogeneity. Although sensitivity is higher for the fixed-effects MAGNA methods compared to the random-effects MAGNA methods (likely more power due to reduced model complexity), specificity severely drops with larger levels of cross-study heterogeneity. This indicates that aggregating over datasets without taking cross-study heterogeneity into account can lead to severely false conclusions in which the false inclusion rate is much higher than the expected $\alpha = 0.05$ and can even rise above 0.50. This marks a distinct need for cross-study heterogeneity to be taken into account.
- **Random-effects MAGNA without cross-study heterogeneity** Figure 2 shows that random-effects MAGNA estimation performs well across the board in retrieving the pooled network structure when there is no cross-study heterogeneity: all three measures are near 1. This marks an interesting comparison to fixed-effects MAGNA: random-effects MAGNA seems to perform even better than fixed-effects MAGNA, as the false inclusion rate is lower. However, this is not entirely accurate, as specificity should be at 0.95 and should not be expected to be higher. As such, the random-effects MAGNA model seems to be too conservative in this setting. It could be that the sparse nature of the true underlying model plays a role in the strong performance in correlation and sensitivity, and that random-effects MAGNA would perform less well with more complex network structures. Figure 3 shows furthermore that the average bias of estimated random effect standard deviations does not go to zero with larger sample sizes as should be expected. To this end, it seems that random-effects MAGNA will always estimate some level of heterogeneity (around 0.3–0.5 on average). This average bias rarely was higher than 0.6.
- **Random-effects MAGNA with cross-study heterogeneity** In the conditions where cross-study heterogeneity was included, Figs. 2 and 3 show that random-effects MAGNA converges to desirable properties with increasing numbers of included studies, as should be expected: the sensitivity and correlation go to 1, the specificity goes to 0.95, and the bias goes to 0. At low numbers of studies, in particular the condition with 4 studies, the sensitivity and correlation are only around 0.7 and the specificity is a bit lower than the expected level of 0.95. While the performance is not bad in this condition, this should be taken into account in empirical applications, and a larger number of studies (e.g., 16 or more) is recommended for applied research.

## Empirical Applications: Meta-analytic Network Models for PTSD Networks

In this section, we show two empirical applications of MAGNA analysis in multiple datasets of PTSD symptoms. The first is a fully reproducible example of MAGNA analysis on a set of four datasets on PTSD symptoms (Fried et al. 2018), and the second is a description of a large-scale meta-analysis using MAGNA, which we describe in more detail elsewhere (Isvoranu et al. in press) Supplement 4 furthermore shows a second empirical example on a homogeneous set of datasets studying anxiety, depression and stress symptoms (Lovibond and Lovibond 1995), obtained from the Open Source Psychometrics Project (openpsychometrics.org). All data and code to reproduce the two empirical examples are available in our online supplementary materials on the Open Science Framework.^13^

### Empirical Example: 4 Datasets of PTSD Symptoms

To illustrate the functionality of MAGNA, we make use of the materials made available by Fried et al. (2018) in their cross-cultural multisite study of PTSD symptoms. The study estimated regularized partial correlation networks of 16 PTSD symptoms across four datasets of traumatized patients receiving treatment for their symptoms. The data were collected in the Netherlands and Denmark, resulting in a total of $n = 2{,}782$ subjects. The first sample consisted of 526 traumatized patients from a Dutch mental health center specializing in treatment of patients with severe psychopathology and a history of complex traumatic events. The second sample consisted of 365 traumatized patients from a Dutch outpatient clinic specializing in treatment of anxiety and related disorders encompassing various trauma types. The third sample consisted of 926 previously deployed Danish soldiers receiving treatment for deployment-related psychopathology at the Military Psychology Clinical within the Danish Defense or were referred for treatment at specialized psychiatric clinical or psychologists in private practice. Finally, the fourth sample consisted of 956 refugees with a permanent residence in Denmark, diagnosed with PTSD and approximately $30\%$ suffered from persistent trauma-related psychotic symptoms.

The Harvard Trauma Questionnaire (HTQ; Mollica et al. 1992) was used to assess symptomatology in samples 1 and 4, the Posttraumatic Stress Symptom Scale Self-Report (PSS-SR; Foa et al. 1997) was used to assess symptomatology in sample 2, and the Civilian version of the PTSD checklist (PCL-C; Weathers et al. 1993) was used to assess symptomatology in sample 3. All instruments were Likert-type scales ranging from 1 to 4 or from 1 to 5. The PCL-C and PSS-SR measured 17 items rather than 16 (i.e., physiological and emotional reactivity symptoms were measured separately). To match the number of items to the HTQ, Fried et al. (2018) combined the two items and used the highest score on either of the two in the analyses.

#### Single-Group Analyses

**Fig. 4:**
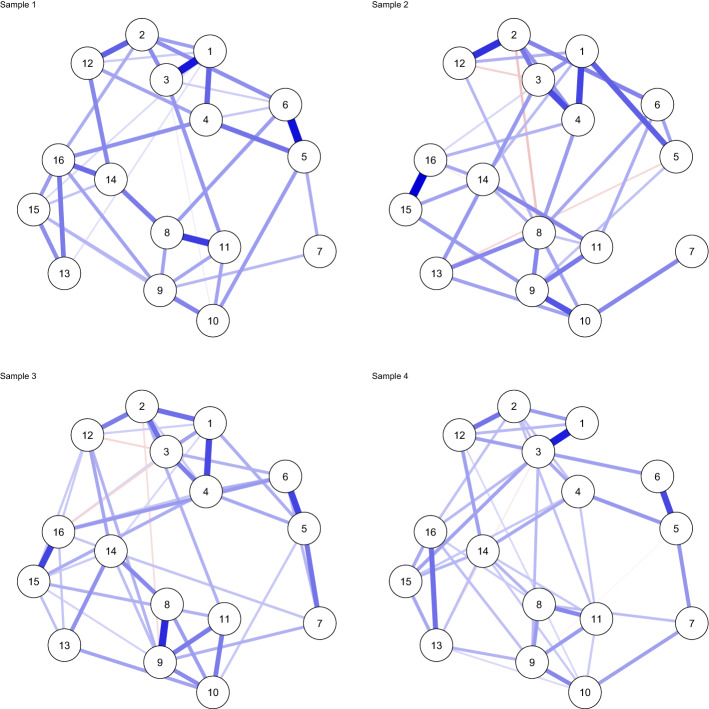
Gaussian graphical model (GGM; a network of partial correlations) structures estimated for each of the four correlation matrices individually ($n_1 = 526$, $n_2 = 365$, $n_3 = 926$, and $n_4 = 956$), reported by (Fried et al. 2018), using significance pruning at $\alpha = 0.05$. Node are (1) intrusions, (2) nightmares, (3) flashbacks, (4) physiological/psychological reactivity, (5) avoidance of thoughts, (6) avoidance of situations, (7) amnesia, (8) disinterest in activities, (9) feeling detached, 10) emotional numbing, (11) foreshortened future, (12) sleep problems, (13) irritability, (14) concentration problems, (15) hypervigilance, and (16) startle response. Blue edges indicate positive partial correlations and red edges indicate negative partial correlations.

Even though Fried et al. (2018) based their main analyses on polychoric correlation matrices because the data were assessed on a likert scale, we make use of the Pearson correlation matrices provided in their supplementary materials. We do this because polychoric correlations do not evaluate to the likelihood of the data, and because polychoric correlations have been found to be quite unstable in network estimation at lower sample sizes (Fried et al. 2021) We estimated a GGM using significance pruning for each dataset individually in the same way as described in Sect. 3.2, Supplement 1 and Supplement 2.1. The results can be seen in Fig. 4, which shows that the estimated structures are quite similar but also differ in several ways.

#### Multi-dataset Analysis: Fixed-Effects MAGNA and Partial Pruning

**Fig. 5:**
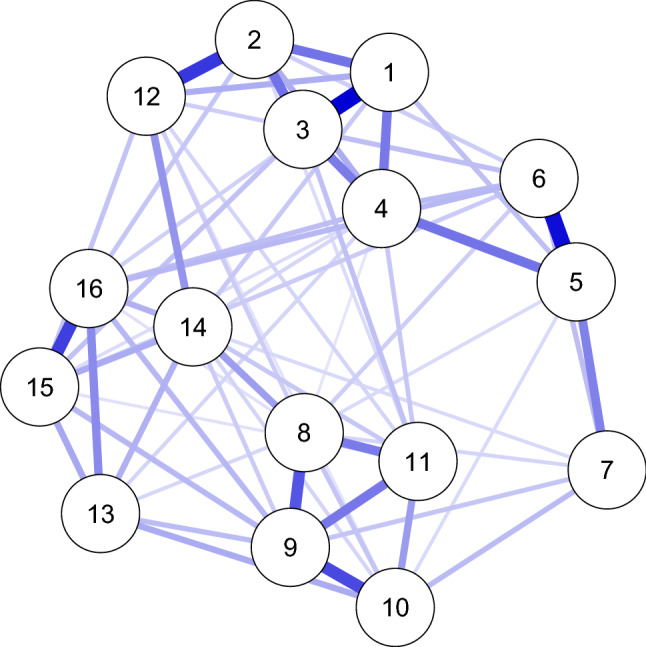
Pooled GGM structure estimated with multi-dataset fixed-effects MAGNA over all four correlation matrices reported by Fried et al. (2018), using significance pruning at $\alpha = 0.05$. Node descriptions are given in Fig. 4.

**Fig. 6:**
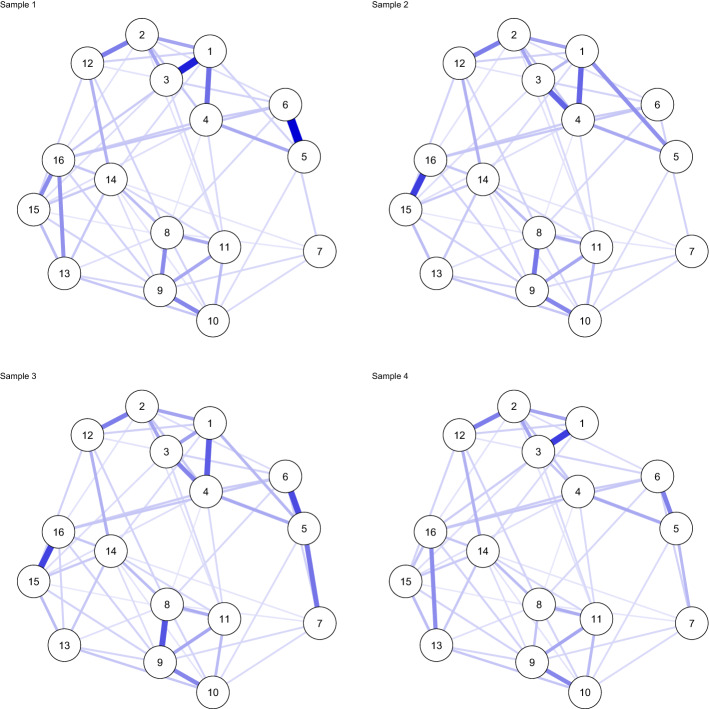
Partially pooled GGM estimates for correlation matrices reported by Fried et al. (2018). Network structures were estimated by first fitting a pooled model (Fig. 5) and subsequently using a stepwise search algorithm that searches for differences between groups. Node descriptions are given in Fig. 4.

Continuing the example, we used a multi-dataset approach to estimate a pooled GGM over all four correlation matrices reported by Fried et al. (2018). Figure 5 shows the resulting network structure. Of note, however, is that the model with a fixed structure over all four groups fits worse than the model in which each group has a unique GGM structure (Fig. 4) in terms of AIC (112,017 versus 110,809 for the unique model) and BIC (112,414 versus 111,776 for the unique model). The model also did not fit very well according to RMSEA (0.074) while the unique model fitted much better (0.035). To this end, we also estimated a *partially pruned* model as described in Sect. 4.2.3. In the partially pruned model, shown in Fig. 6, out of 120 potential edges, 49 were included and constrained equal across all datasets, 61 were set to zero in all datasets, 3 edges were included in all datasets but not equal across datasets (1 – 3, 8 – 9, and 15 – 16), and 7 edges were included in some but not all datasets (1 – 4, 3 – 4, 1 – 5, 5 – 6, 5 – 7, 1 – 16, and 13 – 16). The partially pruned model had a good fit according to RMSEA (0.041), and fitted best in terms of BIC (111,390) but not AIC (110,916). Here it should be noted, however, that the algorithm used for partial pruning is very exploratory and designed to optimize BIC.

**Fig. 7:**
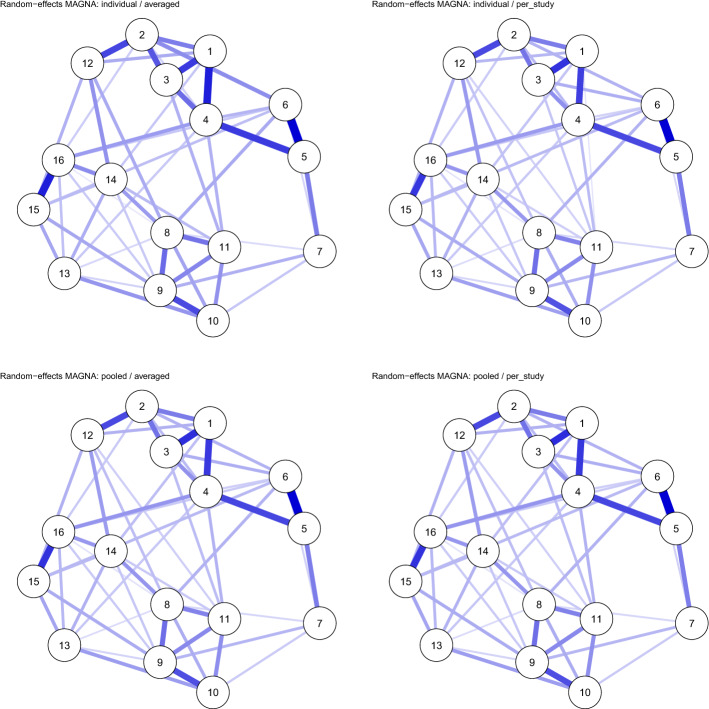
Estimated random-effects MAGNA GGM structures for the correlation matrices reported by Fried et al. (2018). Node descriptions are given in Fig. 4.

**Fig. 8:**
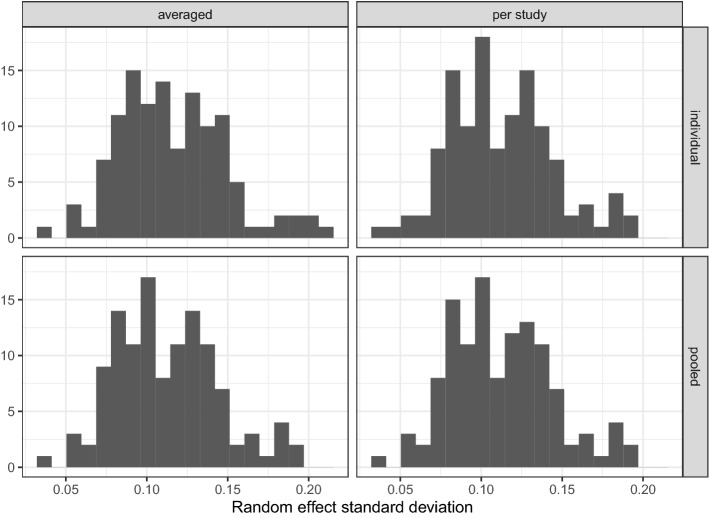
Estimated standard deviations of random effects for the correlation matrices reported by Fried et al. (2018)

#### Random-Effects MAGNA

Finally, we performed random-effects MAGNA estimation using all four estimation variants discussed above. The resulting common GGM structures can be seen in Fig. 7, and the random effect standard-deviation estimates can be seen in Fig. 8. It can be noted that the estimated GGMs are much sparser than the unique and (partially) pooled GGMs estimated earlier. This is likely due to a far more complex model setup (7,260 added parameters) in combination with a small sample size (only four sets of correlations). Nonetheless, the overall structure remains similar. The random effect estimates vary mostly between about 0.06 and 0.17 and were on average 0.11, which marks large random effect sizes on the correlational structure; the simulation studies in Sect. 6 consider at most a standard deviation of 0.1, and show that in a fully homogeneous setting estimated random effect standard deviations would on average not exceed 0.6.

### Empirical Application: A Meta-analysis of 52 PTSD Datasets

Given that carrying out and describing a complete meta-analysis in this paper would not be feasible due to space constraints, we restricted empirical examples in the current paper to the four datasets described in Sect. 7.1 and the depression, anxiety and stress symptom analysis in Supplement 4. However, it should be noted that four datasets is a low number of studies for meta-analytic purposes. Our simulations showed that a 16-node model could be estimated from four datasets, but also that we should expect lower sensitivity (lower statistical power), a lower specificity than desired (some included false edges), and biased estimates of the random-effect standard deviations. To this end, the results shown in Fig. 7 should be seen more as an exemplification of the method rather than a substantive contribution to the PTSD literature. Recently, we have also applied MAGNA analysis to a larger set of 52 datasets used to study PTSD symptom networks, which we obtained through a systematic search (Isvoranu et al. in press) Here, we highlight the main findings of this study; we refer to (Isvoranu et al. in press) for more details on this meta-analysis.

First, the common network structure identified in the meta-analytic work was relatively dense, with strong connections between symptoms. The strongest associations were identified between the symptoms “hypervigilant” and “easily startled”, “internal avoidance” and “external avoidance”, “emotional numbing” and “feeling detached”, as well as “feeling detached” and “loss of interest”. These results aligned well with results from a previously reported review on PTSD symptom network (Birkeland et al. 2020), with a recent analysis of a large sample of veterans (Duek et al. 2021), as well as with the example described in Sect. 7.1, which analyzes a subset of the larger dataset discussed in (Isvoranu et al. in press)

In terms of centrality—descriptive statistics often used in network analysis in attempts to identify the most influential nodes (Bringmann et al. 2019)—the extended meta-analysis identified especially the symptoms “feeling detached”. “intrusive thoughts,” and “physiological reactivity” to be higher than other symptoms on measures of direct centrality and the symptoms “nightmares” and “sleep disturbance” to be higher than other symptoms on measures of indirect centrality. Furthermore, the symptom “amnesia” was consistently the least central symptom. Of note, however, was that differences in these centrality indices were very small.

Second, in line with the results from the example in Sect. 7.1, the extended meta-analysis identified large cross-study heterogeneity, with large random effect sizes on the correlation structure. The extended meta-analysis reported even higher random effect standard deviations than the example in Sect. 7.1, with estimates ranging from 0.10 to 0.18. These results indicate that populations may differ in their underlying network structure and that expecting one single study to fully recover a generalizable structure is not feasible. Nonetheless, and of note, further investigations identified good correspondence between network models estimated from single datasets to the network model estimated using MAGNA, with regularization techniques leading to more generalizable network parameters and unregularized model search leading to more generalizable network structures.

### Heterogeneity and Replicability of PTSD Networks

In the empirical example described in Sect. 7.1, we analyzed PTSD symptom networks based on four correlation matrices supplied by Fried et al. (2018), and found evidence for heterogeneity across these samples. First, models estimated on each dataset separately differed from one-another on some key aspects. Second, a model with unique networks per dataset fitted better than a fixed-effects MAGNA model in which a pooled GGM was estimated, and even showed comparable fit to a highly exploratory partially pooled GGM model. Finally, random effect standard deviation estimates were estimated to be fairly high (between 0.06 and 0.17 on the correlations). This result is reflected in prior research on these correlational structures: Fried et al. (2018), who compiled the set of correlation matrices, used permutation tests (van Borkulo et al. 2017) and noted several significant differences between estimated GGM structures, Williams et al. (2020) used a Bayesian test and also noted differences in GGM structures, and finally, Forbes et al. (2021) highlighted several more differences between the networks estimated by Fried et al. (2018). While Fried et al. (2018) and Williams et al. (2020) took these findings to represent heterogeneity across groups, Forbes et al. (2021) instead discussed these findings as evidence for ‘poor replicability’ of certain GGM estimation procedures. Our larger meta-analysis, summarized in Sect. 7.2 and further described in (Isvoranu et al. in press) corroborated the finding that heterogeneity across datasets used to study PTSD networks can be expected to be large. Our simulation studies suggest that when heterogeneity is expected to be large, researchers should take this into account when aiming to find a single pooled GGM across studies. Disregarding heterogeneity—whether through fixed-effects MAGNA or other methods such as averaging weight matrices—may lead to a large number of falsely included edges. This also means that, when assessing replicability of network models, not only should one take expected replicability given an estimation method into account (Williams 2020), one should also take potential heterogeneity across datasets into account (Kenny and Judd 2019).

## Discussion

This paper introduced maximum likelihood estimation of Gaussian graphical models (GGMs, networks of partial correlation coefficients) from one or several datasets summarized in correlation matrices. We introduced meta-analytic Gaussian network aggregation (MAGNA), which is based on meta-analytic structural equation modeling (MASEM; Cheung 2015a; Cheung and Chan 2005) and can be used to estimate a common network structure across multiple datasets. In fixed-effects MAGNA, every dataset is assumed to come from the same population, and heterogeneity across datasets is not taken into account. However, there are fixed-effects models that do not assume homogeneity of effect sizes (see Rice et al. 2018). In random-effects MAGNA on the other hand, this heterogeneity (e.g., cultural differences) can be taken into account by modeling a random effect structure on the *correlational* structure. As such, random-effects MAGNA is a multi-level model, although the multi-level component is not placed on the GGM structure itself. To this end, random-effects MAGNA allows for inference about parameters beyond the studies included in the meta-analysis (Hedges and Vevea 1998). We assessed the performance of MAGNA in large-scale simulation studies, which showed good performance across all methods, although fixed-effects MAGNA performed poorly when the true model contained random effects, and random-effects MAGNA performed best with at least 16 datasets. Finally, we described two empirical applications in the main text, and a third application in supplementary materials. First, we re-analyzed four correlation matrices supplied by Fried et al. (2018) on post-traumatic stress disorder (PTSD) symptoms, and found evidence for heterogeneity across these four groups. Second, we summarized results from a larger meta-analysis on PTSD symptoms (Isvoranu et al. in press) which also showed large cross-study heterogeneity while also showing similar results to earlier non-meta-analytic work (Birkeland et al. 2020; Fried et al. 2018). We discussed implications of this high level of heterogeneity for network aggregation methods and reproducibility of network structures. The supplementary materials include a theoretical tutorial on maximum likelihood estimation, a practical tutorial on how the analyses introduced in this paper can be performed in R, a description of multi-dataset Ising model estimation, and a second empirical example on depression, anxiety and stress symptoms. All methods proposed in this paper are implemented in the freely available software package *psychonetrics* (Epskamp 2020a; b), and reproducible code for the empirical examples can be found on the Open Science Framework.$^{13}$

**Limitations**

Several limitations to the current work should be mentioned. First, especially random-effects MAGNA is computationally challenging, and the current implementation in *psychonetrics* can be very slow and can require a lot of resources. All empirical examples were run on a computer with an AMD Ryzen 9 3950X processor, a RTX 2080 Ti GPU, and 128 GB of 3600MHz DDR4 RAM, and the simulation study was run on a high-performance cloud system. The empirical example reported in Supplement 4 consisted of 21 nodes, which was the most challenging to estimate. Adding many more nodes to MAGNA analyses currently may not be feasible in practice. A second important limitation is the assumption that all correlation matrices summarize fully observed multivariate normally distributed data. Most applications of GGM models, however, are seen in clinical datasets that are usually measured on ordered categorical scales, such as the scales used in the PTSD examples. In addition, missing data is also prevalent in clinical datasets. To this end, it is questionable how severe violations of these assumptions are. With regard to violations of normality, Spearman correlation matrices may potentially be used instead of Pearson correlation matrices (as Spearman correlations are simply Pearson correlations on the rank), and future research could investigate the performance of such measures. With regard to missing data, pairwise estimated correlation matrices, such as provided by Fried et al. (2018), could be used instead. However, care needs to be taken then in setting the number of observations. Fried et al. (2018) reported only low amounts of missing data, and as such we used the sample sizes provided in the paper in the analyses, even though the correlation matrices were estimated pairwise. An alternative is to use the average sample size available for each individual correlation, or the minimum sample size of participants that had no missings. Of note, when raw datasets are available, single dataset and fixed-effects MAGNA estimation can readily be done using full-information maximum likelihood (FIML) estimation, which is supported in *psychonetrics*. FIML cannot be used to handle missing data in random-effects MAGNA, however, although it could be used to handle missing *variables* in datasets—a feature that is implemented in *psychonetrics* as well.

**Future directions and related work** While this paper only discussed estimation of correlation matrices, GGMs, and the Cholesky decomposition, the presented framework in Sect. 2—which lies at the core of the *psychonetrics* package—is much more flexible. Other Gaussian models can readily be estimated by changing only the *model Jacobian*. Beyond GGM estimation, *psychonetrics* now contains such modeling frameworks for structural equation models (Bollen and Stine 1993), latent/residual network models (Epskamp et al. 2017), graphical vector-auto regressive models (Epskamp et al. 2018), and dynamical factor models (Epskamp, 2020a). Beyond Gaussian models, implementing different *distribution Jacobian*s and Hessians allows for other modeling frameworks as well, such as the dichotomous Ising models (Marsman et al. 2018) further explained in Supplement 5. All these models are implemented for multi-dataset specification and allow for estimating fixed-effects estimation across datasets. Future research could focus on the performance of multi-dataset models from the above-mentioned frameworks.

With regards to model selection, we only investigate significance pruning (removing edges at a fixed $\alpha$ and re-evaluating the model) in this paper because (a) it is the fastest and most basic algorithm available, and (b) it allows us to evaluate if expected properties of significance testing hold (e.g., specificity should equalize at $1 - \alpha$; Williams and Rast, 2018). More advanced model selection algorithms may be utilized to investigate the network structure. In GGM estimation, a popular technique is to use regularization (Epskamp and Fried 2018), although it has recently been shown that such regularization techniques are no longer beneficial at sufficient sample sizes (Williams and Rast 2018). To this end, non-regularized model estimation has grown more popular, especially in larger datasets (Isvoranu et al. 2019; Kan et al. 2019; Williams et al. 2019). Examples of such non-regularized algorithms include the ggmModSelect implemented in *qgraph* (Epskamp et al. 2012) and several algorithms implemented in the *GGMnonreg* package (Williams et al. 2019). The *psychonetrics* package does not include regularized GGM estimation, but does include more advanced non-regularized estimation methods through recursive pruning with the prune function, step-up model search with the stepup function, and extensive stepwise search through the modelsearch function (Epskamp, 2020a). These algorithms could also be applied to MAGNA estimation, although arguments can also be made that such more advanced model search algorithms could hamper inference (Leeb and Pötscher 2005; Leeb et al. 2006). In principle, regularized model search strategies could also be applied to estimating a pooled network structure across datasets, for example by employing regularization techniques that search for similarity across datasets (Costantini et al. 2019). However, it should also be noted that precise inference, such as the fixed false-positive rates demonstrated in the MAGNA methods, are far from trivial using regularization techniques (Jankova et al. 2015; Javanmard and Montanari 2014; Van de Geer et al. 2014).

Further future directions involve additional extensions of meta-analytic capacities in network psychometrics. Currently, only meta-analytic random effect models are implemented for variance–covariance based models (e.g., GGM, Cholesky decomposition, and a general variance–covariance matrix) in *psychonetrics*, but general MASEM models are implemented in the *metaSEM* package (Cheung 2015b). A future direction would be to also implement meta-analytic random effect variants of the above discussed models in *psychonetrics*. Finally, recent work in meta-analytic SEM allows for study-level moderation effects to be included (Jak and Cheung 2020), which can potentially also be extended to GGMs as well.

## Conclusion

This paper introduced methods for estimating network models while aggregating over multiple datasets. To this end, this paper opens the door for meta-analytic research in network psychometrics, which given the popularity of these models in recent literature may be warranted (Robinaugh et al. 2020). Given that network models contain many parameters, the ability to aggregate over multiple datasets may prove vital for the further maturation of the field.

### Supplementary Information

Below is the link to the electronic supplementary material.

Supplementary material 1 (pdf 523 KB)

## Appendix: Vectorization Matrices

Throughout this paper, we make use of several sparse matrices.

- The *duplication matrix* $\pmb {D}$ can be used to transform between (column-stacked) vectorized matrices and half vectorized matrices for symmetric matrices. That is, $\pmb {D}$ is the solution to:
  $$\begin{aligned} \mathrm {vec}\left( \pmb {X}\right) = \pmb {D} \, \mathrm {vech}\left( \pmb {X}\right) . \end{aligned}$$
  We will also define $\pmb {D}^*$ for square symmetric matrices that have zeroes on the diagonal:
  $$\begin{aligned} \mathrm {vec}\left( \pmb {X}\right) = \pmb {D}_{*} \, \mathrm {vechs}\left( \pmb {X}\right) . \end{aligned}$$
- The *elimination matrices* $\pmb {L}$ and $\pmb {L}_{*}$ allow for the inverse transformations:
  $$\begin{aligned} \mathrm {vech}\left( \pmb {X}\right)&= \pmb {L} \, \mathrm {vec}\left( \pmb {X}\right) \\ \mathrm {vechs}\left( \pmb {X}\right)&= \pmb {L}_{*} \, \mathrm {vec}\left( \pmb {X}\right) . \end{aligned}$$
  It can be noted that $\pmb {D} \pmb {L} = \pmb {I}$.
- The *diagonalization matrix* $\pmb {A}$ is a permutation matrix that can be used for transformations between the vectorized form and the diagonal of a diagonal matrix:
  $$\begin{aligned} \mathrm {vec}(\pmb {X})&= \pmb {A} \,\mathrm {diag}(\pmb {X}) \\ \mathrm {diag}(\pmb {X})&= \pmb {A}^{\top } \, \mathrm {vec}(\pmb {X}). \end{aligned}$$
- The *commutation matrix* $\pmb {C}$, is a permutation matrix that can be used to transform between the vectorized form of an asymmetric matrix and the vectorized form of the transpose of that matrix:^14^
  $$\begin{aligned} \mathrm {vec}(\pmb {X}^{\top })&= \pmb {C} \mathrm {vec}(\pmb {X})\\ \mathrm {vec}(\pmb {X})&= \pmb {C}^{\top } \mathrm {vec}(\pmb {X}^{\top })\\ \end{aligned}$$

Functions for these matrices are implemented in the *lavaan* and *psychonetrics* packages.

## Footnotes

1 Of note, we term this analysis method *multi-dataset* analysis in this paper, but the term *multi-group* analysis is also commonly used in, for example, structural equation modeling (SEM; Bollen and Stine 1993).

2 Of note: this equation is a matrix form of obtaining partial correlation coefficients through standardizing the inverse variance-covariance matrix and multiplying all elements by $-1$, a relationship that is well known and can be traced back as far as the work of Guttman (1938).

3 Version 0.9 of the *psychonetrics* package has been used for all analyses in this paper, using the *nlminb* optimizer with a relative tolerance of $\sqrt{\epsilon } \approx 1.49 \times 10^{-8}$ (the square root of the smallest possible positive floating point number). This is a conservative optimization technique that can be slow to run: the empirical example took several hours to run on a computer with an AMD Ryzen 9 3950X processor, a RTX 2080 Ti GPU, and 128 GB of 3600MHz DDR4 RAM. For faster results, a different optimizer or a lower tolerance level can be used.

4 While multiplying the log-likelihood with $-2 / n$ is not required, we do this as it is common practice, because it simplifies notation, and to not have the fit function depend on *n* in the optimization routine.

5 The *psychonetrics* package makes use of the optimization routine implemented in the R function nlminb.

6 For the analyses in Sects. 3 and 4.2 data can be missing and handled through full-information maximum likelihood estimation, as explained in Supplement 3. However, we assume no missingness as the aim of this paper is to rely on summary statistics only, and because summary statistics are used as input to the random-effects MAGNA.

7 Throughout this paper we assume the ML estimate is used for standard deviations and variances. That is, the denominator *n* is used rather than the denominator $n-1$.

8 Note that the size of $\pmb {\phi }_i$ and $\pmb {\theta }_i$ may vary over datasets. For example, some datasets may have more variables than other datasets.

9 The implementation of FIML for *psychonetrics* does not split the data per row but rather per block of data with the same missing patterns.

10 Of note, strictly speaking, the fixed-effects MAGNA model should be called a common-effects model, which assumes that the population effect sizes are identical. Some fixed-effects models in the meta analysis literature do not require the assumption of homogeneity of effect sizes (e.g., see Bonett 2009; Rice et al. 2018).

11 It is important to double-check exactly how the WLS weight matrix should be provided. Some software packages that allow for WLS estimation of, for example, structural equation models do not take the Fisher information but rather the parameter variance-covariance matrix as input, with the WLS weight matrix then computed internally. In addition, sample size needs to be assigned in software packages as well, and the manner in which this is done may differ between software packages. In some cases, the sample size could be included in the Fisher information matrix already, in which case a sample size of 1 should be given to software. If the unit Fisher information matrix from Eq. (5) is used, the sample size needs to be given explicitly, as is the case in the *psychonetrics* package. Supplement 2.2.1 gives an example of how to properly assign the weight matrix using the *psychonetrics* package.

12 Of note, the symbols $\pmb {\mu }_i$ and $\pmb {\Sigma }_i$ are often used to denote the mean and variance-covariance structure of the responses $\pmb {y}$ rather than the sample correlation coefficients. Here, they specifically indicate the structure of sample correlation coefficients.

13 https://www.osf.io/67bxd/.
