# Supplementary material for: Meta-analytic Gaussian Network Aggregation
Source: Psychometrika. 2021 Jul 15;87(1):12–46. doi: 10.1007/s11336-021-09764-3 (PMC9021114; doi:10.1007/s11336-021-09764-3)
Supplement: Supplementary file 1 — Supplementary material 1 (pdf 523 KB) [file 11336_2021_9764_MOESM1_ESM.pdf]

Meta-analytic Gaussian Network Aggregation Supplementary Materials

Sacha Epskamp[1,2], Adela-Maria Isvoranu[1], and Mike W.-L. Cheung[3]

[1]Department of Psychology, University of Amsterdam
[2]Centre for Urban Mental Health, University of Amsterdam
[3]Department of Psychology, National University of Singapore

Contents

1 GGM Estimation Example **2**

2 A Tutorial on GGM Estimation using *psychonetrics* **3**
2.1 Single Dataset Estimation 4
2.2 Multiple Dataset ML Estimation: Fixed-effects MAGNA 5
2.2.1 Two-stage Estimation 6
2.2.2 Multi-dataset Estimation 7
2.3 Multiple Dataset ML Estimation: Random-effects MAGNA 8

3 Full-information Maximum Likelihood **9**

4 Empirical Example: Depression, Anxiety and Stress **10**

5 Estimating Multi-group Ising Models from Summary Statistics **13**

1 GGM Estimation Example

In this supplement, we will detail the steps used in maximum likelihood estimation of a Gaussian graphical model (GGM) for a small example. Suppose that a dataset of three variables with 1,000 observations gives the following sample correlation matrix:

$$\boldsymbol{R} = \begin{bmatrix} 1 & 0.31 & -0.09 \\ 0.31 & 1 & -0.23 \\ -0.09 & -0.23 & 1 \end{bmatrix}.$$

Given that there are three variables, there are $3 \cdot 2/2 = 3$ potential parameters in the GGM, leading to the model parameters $\boldsymbol{\theta}^\top = \begin{bmatrix}\omega_{21} & \omega_{31} & \omega_{32}\end{bmatrix}$. We first wish to estimate a saturated GGM in which all parameters are included. The manual Jacobian then becomes an identity matrix:

$$\frac{\partial \boldsymbol{\theta}}{\partial \boldsymbol{\psi}} = \begin{matrix} \\ \omega_{21} \\ \omega_{31} \\ \omega_{32} \end{matrix} \begin{matrix} \begin{matrix} \psi_1 & \psi_2 & \psi_3 \end{matrix} \\ \begin{bmatrix} 1 & 0 & 0 \\ 0 & 1 & 0 \\ 0 & 0 & 1 \end{bmatrix} \end{matrix}.$$

This matrix encodes a model in which the free parameters (parameters to be estimated) are $\psi_1 = \omega_{21}$, $\psi_2 = \omega_{31}$, and $\psi_3 = \omega_{32}$. Next, we could estimate the parameters by minimizing Equation (2) (finding a solution on which Equation (3) is a vector of zeroes). For the saturated model, however, we don't have to perform iterative model search and can obtain ML estimates directly by standardizing the inverse of $\boldsymbol{R}$ and multiplying off-diagonal elements with -1 (Epskamp, Waldorp, et al., 2018; Lauritzen, 1996). This process is automated in, for example, the `cor2pcor` function from the *corpcor* package (Schafer et al., 2017). The ML estimate becomes:

$$\widehat{\boldsymbol{\Omega}} = \begin{bmatrix} 0 & 0.30 & -0.02 \\ 0.30 & 0 & -0.21 \\ -0.02 & -0.21 & 0 \end{bmatrix}.$$

With this estimate, we can compute the parameter variance-covariance matrix in Equation (6). The square root of the diagonal of this matrix gives the standard errors 0.028, 0.032, and 0.030, which can be used to obtain the p-values in order to test whether each edge is non-zero:

$$\begin{matrix} \omega_{21} \\ \omega_{31} \\ \omega_{32} \end{matrix} \begin{bmatrix} < 0.01 \\ 0.52 \\ < 0.01 \end{bmatrix}$$

The edge between the first and the third variable is not significant. Next, we could fit a model with this edge constrained to zero. To do this, we specify the manual Jacobian

as:

$$\frac{\partial \boldsymbol{\theta}}{\partial \boldsymbol{\psi}} = \begin{array}{c} \\ \omega_{21} \\ \omega_{31} \\ \omega_{32} \end{array} \begin{array}{c} \begin{array}{cc} \psi_1 & \psi_2 \end{array} \\ \begin{bmatrix} 1 & 0 \\ 0 & 0 \\ 0 & 1 \end{bmatrix} \end{array}.$$

This model encodes that we now have two parameters to estimate: $\psi_1 = \omega_{21}$ and $\psi_2 = \omega_{32}$. As there is no free parameter for the edge ω_{31}, this parameter is no longer included in the model and its value will be fixed to its starting value (in this case, 0). All other Jacobians have the same form as before (although numeric values will differ). Next, we can fix this element to zero in a matrix of starting values for $\boldsymbol{\Omega}$, and we can numerically optimize Equation (2). We then obtain as estimates:

$$\widehat{\boldsymbol{\Omega}} = \begin{bmatrix} 0 & 0.30 & 0 \\ 0.30 & 0 & -0.22 \\ 0 & -0.22 & 0 \end{bmatrix}.$$

Note that these estimates are slightly different also for other edges in the model. We will term this process of removing non-significant edges and re-estimating parameters *pruning*. This is the most basic form of model selection of network models.[1]

2 A Tutorial on GGM Estimation using *psychonetrics*

In this supplement, we describe how the *psychonetrics* package (Epskamp, 2020a, 2020b) for the statistical programming language *R* (R Core Team, 2019) can be used for performing the analyses described in the paper. For the single-group models there are many other software packages that estimate pruned GGMs in which some edges are set to equal zero. For example, the *glasso* (Friedman et al., 2019; Friedman et al., 2008) package can be used to estimate a GGM for a given network structure (the regularization parameter needs to be set to zero), which is utilized in the `ggmFit` function in the *qgraph* package (Epskamp et al., 2012). Furthermore, the *GGMnonreg* (Williams et al., 2019) can be used for ML pruning methods. More advanced ML model search strategies have also been implemented in *qgraph* (the `ggmModSelect` routine) and *GGMnonreg*. Finally, Bayesian estimation procedures have been implemented in the *BDgraph* (Mohammadi & Wit, 2019) and *BGGM* (Williams & Mulder, 2020) packages.

First, we need to install the *psychonetrics* package:[2]

```
install.packages("psychonetrics")
```

Next, we can load the *psychonetrics* package as well as the *qgraph* package for drawing networks and the *dplyr* (Wickham et al., 2019) package to gain access to the pipe operator `%>%`:

[1] For more advanced options that utilize the estimated Fisher information matrix as discussed in this paper, see (Epskamp, 2020a).

[2] Alternatively, the developmental version can be installed from Github with the command `devtools::install_github("sachaepskamp/psychonetrics")`.

```
library("psychonetrics")
library("dplyr")
library("qgraph")
```

The pipe operator allows us to write `f(x,...)`as `x %>% f(...)`, in which `x` is some object, `f` some function, and `...` any number of arguments. This notation simplifies reading R code, as code can now be read from left to right rather than from inside to outside.

2.1 Single Dataset Estimation

Suppose we have the correlation matrix described above:

```
corMat <- matrix(
    c(1, 0.31, -0.09,
    0.31, 1, -0.23,
    -0.09, -0.23, 1),
  nrow = 3, ncol = 3, byrow=TRUE)
```

and that this is based on $n = 1{,}000$ observations:

```
nobs <- 1000
```

In *psychonetrics*, we first need to form a model object, which is subsequently evaluated and adjusted. The `ggm` function can be used to form a GGM model:

```
mod1 <- ggm(
  covs = corMat,
  nobs = nobs,
  corinput = TRUE
)
```

The command above forms a model based on a correlation matrix as input: the `covs` argument takes a single covariance (or correlation) matrix or a list of such matrices, the `nobs` argument takes a single number of observations or a vector for each dataset, and the `corinput` argument tells *psychonetrics* that the input is a correlation matrix. By default, a model is formed in which all edges are included. For fitting a pre-specified GGM structure, the `omega` argument could be supplied a binary matrix with a 1 indicating a parameter is free and a 0 indicating a parameter is fixed to zero. Next, we can run the model:

```
mod1 <- mod1 %>% runmodel
```

Finally, we can investigate the parameters:

```
mod1 %>% parameters
```

which gives as output:

```
-  omega (symmetric)
 var1 op var2    est    se        p row col par
   V2 --   V1   0.30 0.028 < 0.0001   2   1   1
   V3 --   V1 -0.020 0.032     0.52   3   1   2
   V3 --   V2  -0.21 0.030 < 0.0001   3   2   3
```

Here, the `var1`, `op`, and `var2` columns indicate what type of parameter is estimated, the `est` column indicates the ML estimate, the `se` column the standard error, the `p` column the *p*-value (which slightly differs from above due to rounding), the `row` and `col` columns the row and column index of the matrix, and finally the `par` column the parameter number (e.g., row and column index of the Fisher information matrix). To fit a model in which the edge `V3 -- V1` is fixed to zero we can use the `fixpar` command:

```
mod2 <- mod1 %>% fixpar("omega","V1","V3") %>% runmodel
```

Alternatively, we could have used the `prune` function to automate this:

```
mod2 <- mod1 %>% prune(alpha = 0.05)
```

We can compare these models using the `compare` function:

```
compare(
  saturated = mod1,
  pruned = mod2
)
```

Which gives:

```
     model DF     AIC     BIC RMSEA Chisq Chisq_diff DF_diff p_value
 saturated  0 8363.84 8378.56
    pruned  1 8362.24 8372.06   ~ 0  0.41       0.41       1    0.52
```

The output includes the AIC, BIC, and a χ^2 difference test (which is only valid if the models are nested). All these measures indicate that the pruned model fits better. Finally, to obtain the network structure, we can use the `getmatrix` command:

```
net <- getmatrix(mod2, "omega")
```

which can subsequently be plotted using *qgraph*:

```
qgraph(net, theme = "colorblind", layout = "spring")
```

2.2 Multiple Dataset ML Estimation: Fixed-effects MAGNA

Suppose we observed two more correlation matrices based on samples of 500 cases each:

```
corMats <- list(
  matrix(
    c(1, 0.31, -0.09,
      0.31, 1, -0.23,
      -0.09, -0.23, 1),
  nrow = 3, ncol = 3),

  matrix(c(
    1, 0.27, -0.01,
    0.27, 1, -0.18,
    -0.01, -0.18, 1),
  nrow = 3, ncol = 3),

  matrix(c(
    1, 0.23, -0.01,
    0.23, 1, -0.25,
    -0.01, -0.25, 1),
  nrow = 3, ncol = 3)
)

nobs <- c(1000, 500, 500)
```

2.2.1 Two-stage Estimation

In two-stage estimation, we first fit a model for a common correlation matrix over all datasets. The model can be formed with the `corr` function:

```
mod_stage1 <- corr(
  covs = corMats,
  nobs = nobs,
  corinput = TRUE,
  equal = "rho"
) %>% runmodel
```

Setting the `equal` command to `"rho"` ($\boldsymbol{P}$) tells *psychonetrics* we wish to fit an equal correlational structure (alternatively, the `groupequal` command could have been used after forming the model). Typical SEM fit indices can be obtained to check if this pooled correlational structure fits well:

```
mod_stage1 %>% fit
```

The output shows excellent fit. Next, we can obtain the pooled correlational structure:

```
pool_cors <- mod_stage1 %>% getmatrix("rho",group = 1)
diag(pool_cors) <- 1
```

as well as the Fisher information matrix:

```
Fisher <- mod_stage1@information
```

Which can subsequently be used for the stage two model:

```
mod_stage2 <- ggm(
  covs = pool_cors,
  nobs = sum(nobs),
  corinput = TRUE,
  estimator = "WLS",
  WLS.W = Fisher
) %>% runmodel
```

The `estimator = "WLS"` argument tells *psychonetrics* to use WLS estimation, and the `WLS.W` argument sets the WLS weights matrix. This model can be used in exactly the same way as the model described in section 2.1. For example, we can investigate parameter estimates with:

```
mod_stage2 %>% parameters
```

Which gives:

```
-  omega (symmetric)
 var1 op var2   est    se        p row col par
   V2 --   V1  0.28 0.020 < 0.0001   2   1   1
   V3 --   V1 0.013 0.022     0.56   3   1   2
   V3 --   V2 -0.22 0.021 < 0.0001   3   2   3
```

2.2.2 Multi-dataset Estimation

For multi-dataset estimation, we can form a common model directly using the `ggm` function, using the same command as above but replacing `corr` with `ggm` and specifying that now the `omega` matrix must be contained equal:

```
mod_common <- ggm(
  covs = corMats,
  nobs = nobs,
  corinput = TRUE,
  equal = "omega"
) %>% runmodel
```

Like before, we can look at the parameter estimates with:

```
mod_common %>% parameters
```

Which shows the exact same estimates as above.

2.3 Multiple Dataset ML Estimation: Random-effects MAGNA

Continuing the example from Section 2.2, we can form the random-effects MAGNA model with the `meta_ggm` function:

```
mod_ranef <- meta_ggm(
  cors = corMats,
  nobs = nobs,
  Vmethod = "individual",
  Vestimation = "per_study"
)
```

Here, the `Vmethod` argument controls how we form the $\boldsymbol{V}_i$ matrix, which can be set to `"individual"` for individual estimation of `"pooled"` for pooled estimation. The `Vestimation` argument controls how the estimator will handle the sampling variation, which can be set to `"averaged"` for averaging over all estimates or `"per_study"` to use unique estimates per study. As before, we can use the `runmodel` and `prune` commands to run and prune the model:

```
mod_ranef <- mod_ranef %>% runmodel  %>% prune(alpha=0.05)
```

We can again investigate the parameters with:

```
mod_ranef %>% parameters
```

which gives (truncated):

```
     -  omega_y (symmetric)
 var1 op var2   est    se        p row col par
   V2 --    V1  0.27 0.022 < 0.0001   2   1   1
   V3 --    V2 -0.21 0.020 < 0.0001   3   2   2
```

showing that the same edge as before was removed. All the elements of $\boldsymbol{T}$ are estimated to be near zero and non-significant, indicating that there are now random effects. The estimates of the random effect standard deviations can be obtained via:

```
mod_ranef %>% getmatrix("sigma_randomEffects") %>%
  diag %>%
  sqrt %>%
  round(3)
```

which also show very low estimates:

```
[1] 0.019 0.023 0.002
```

3 Full-information Maximum Likelihood

The log-likelihood function $\mathcal{L}$ will typically take the form of a sum of log-likelihoods for n individual cases:

$$\mathcal{L} = \sum_{c=1}^{n} \mathcal{L}_c.$$

Often, $\mathcal{L}$ will only be a function of summary statistics from the data, and there is no need to explicitly sum over individual likelihoods in the fit function computation. However, there may be reasons to instead define the fit function per individual case. In the main text, we discuss for example that the distribution may be assumed differently per case due to each case representing a set of sample correlations with more or less sampling variation. Another common reason for using a case-wise fit function is when some observations are missing. When using maximum likelihood estimation, the term *full information maximum likelihood* (FIML) estimation is used to describe this optimization scheme. Here, we can define the fit function for each individual case:

$$F = \sum_{c=1}^{n} F_c$$
$$F_c \propto -\frac{2}{n}\mathcal{L}_c.$$

The gradient and Fisher information functions simply take the form of a sum over each case:

$$\boldsymbol{\nabla} F = \sum_{c=1}^{n} \left(\frac{\partial F_c}{\partial \boldsymbol{\psi}}\right)^{\top}$$
$$\boldsymbol{\mathcal{I}} = \sum_{c=1}^{n} \boldsymbol{\mathcal{I}}_c.$$

For each case-wise fit function, we may define a set of case-specific distribution parameters $\boldsymbol{\phi}_c$, which is still a function of a single set of model parameters $\boldsymbol{\theta}$ which in turn is still a function of a single set of free parameters $\boldsymbol{\psi}$. Some datasets may feature missing variables, leading to less distribution parameters to be needed to evaluate the fit function. Let $\boldsymbol{\phi}_c^*$ denote a subset of $\boldsymbol{\phi}_c$ containing only distribution parameters used in the model for case c (e.g., only the means, variances and co-variances related to the variables that are not missing for case c). We can use a filter matrix $\boldsymbol{F}_c$ with zeroes and ones such that:

$$\boldsymbol{\phi}_c^* = \boldsymbol{F}_c \boldsymbol{\phi}_c$$
$$\frac{\partial \boldsymbol{\phi}_c^*}{\partial \boldsymbol{\phi}_c} = \boldsymbol{F}_c.$$

In the special case where no variables are missing, $\boldsymbol{F}_c = \boldsymbol{I}$. We make use of the case-specific distribution set $\boldsymbol{\phi}_c$ and its subset $\boldsymbol{\phi}_c^*$ in two ways:

- We can assume every case is an independent replication of the same model, but not every case has responded to every item. In this case, all case-wise distribution parameters are equal ($\boldsymbol{\phi}_1 = \boldsymbol{\phi}_2 = \ldots = \boldsymbol{\phi}$), but the subsets $\boldsymbol{\phi}^*$ may differ, as different cases may require different subsets of parameters (e.g., a person that did not respond to item 1 does not need the mean of item 1).
- We can also assume that cases are *not* replications from the same model, but rather that a different model generated each of the responses. We do this when modeling sample correlation coefficients to take into account that samples with a low sample size feature less sampling variation than samples with a large sample size.

The case-wise Jacobian takes the same form as Equation (4), with the exception that the *distribution Jacobian* is now defined per case separately and the filter matrix is added:

$$\frac{\partial F_c}{\partial \boldsymbol{\psi}} = \left(\frac{\partial F_c}{\partial \boldsymbol{\phi}_c^*}\right) \boldsymbol{F}_c \left(\frac{\partial \boldsymbol{\phi}_c}{\partial \boldsymbol{\theta}}\right) \left(\frac{\partial \boldsymbol{\theta}}{\partial \boldsymbol{\psi}}\right).$$

Likewise, the case-wise fisher function follows a similar extension:

$$\boldsymbol{\mathcal{I}}_c = \frac{1}{2} \left(\frac{\partial \boldsymbol{\theta}}{\partial \boldsymbol{\psi}}\right)^\top \left(\frac{\partial \boldsymbol{\phi}_c}{\partial \boldsymbol{\theta}}\right)^\top \boldsymbol{F}_c^\top \left(\mathcal{E}\left[\frac{\partial \boldsymbol{\nabla} F_c}{\partial \boldsymbol{\phi}_c^*}\right]\right) \boldsymbol{F}_c \left(\frac{\partial \boldsymbol{\phi}_c}{\partial \boldsymbol{\theta}}\right) \left(\frac{\partial \boldsymbol{\theta}}{\partial \boldsymbol{\psi}}\right).$$

The case-specific *model Jacobian* will take the exact same form as the general *model Jacobian* when FIML is not used. The *distribution Jacobian* and *Distribution Hessian* can be defined per individual case, and usually takes the same form of the derivatives in the general case. No new *manual Jacobian* is required.

4 Empirical Example: Depression, Anxiety and Stress

For a second empirical illustration, we analyzed data of the short version of the Depression Anxiety Stress Scales (DASS21; Lovibond and Lovibond, 1995), which contains 21 items intended to measure depression, anxiety and stress. Table 1 gives a description of the variables included in the DASS21. We obtained the data from the Open Source Psychometrics Project (openpsychometrics.org), which contained n = 39,775 full responses (no missings) on 21 items measured on a 4-point scale. To illustrate MAGNA, we split the data in 40 random samples (average n = 994.3). We estimated a model on the full dataset as described in Section 3, a multi-dataset fixed-effects MAGNA model as described in Section 4.2, and finally a random-effects MAGNA model (individual and averaged estimation) as described in Section 5. As these 'datasets' are drawn from the same population, our expectation is that these results would align. To increase the feasibility of this analysis, we used a slightly lower relative convergence tolerance of 1×10^5.

Figure 1 shows the results of the DASS21 analysis. Panel 1a shows the estimated GGM structures, and Panel 1b the correspondence between edge-weights. The full dataset model and fixed-effects MAGNA model are near identical. The random-effects MAGNA model, on the other hand, is highly similar but sparser than the other models.

Label	Scale	Description
A1	Anxiety	I was aware of dryness of my mouth.
A2	Anxiety	I experienced breathing difficulty (eg, excessively rapid breathing, breathlessness in the absence of physical exertion).
A3	Anxiety	I felt scared without any good reason.
A4	Anxiety	I was aware of the action of my heart in the absence of physical exertion (eg, sense of heart rate increase, heart missing a beat).
A5	Anxiety	I felt I was close to panic.
A6	Anxiety	I was worried about situations in which I might panic and make a fool of myself.
A7	Anxiety	I experienced trembling (eg, in the hands).
D1	Depression	I couldn't seem to experience any positive feeling at all.
D2	Depression	I felt that I had nothing to look forward to.
D3	Depression	I felt I wasn't worth much as a person.
D4	Depression	I felt down-hearted and blue.
D5	Depression	I was unable to become enthusiastic about anything.
D6	Depression	I felt that life was meaningless.
D7	Depression	I found it difficult to work up the initiative to do things.
S1	Stress	I tended to over-react to situations.
S2	Stress	I found it difficult to relax.
S3	Stress	I felt that I was using a lot of nervous energy.
S4	Stress	I felt that I was rather touchy.
S5	Stress	I found it hard to wind down.
S6	Stress	I was intolerant of anything that kept me from getting on with what I was doing.
S7	Stress	I found myself getting agitated.

Table 1: Variable descriptions of the Depression Anxiety Stress Scales (DASS21; Lovibond and Lovibond, 1995).

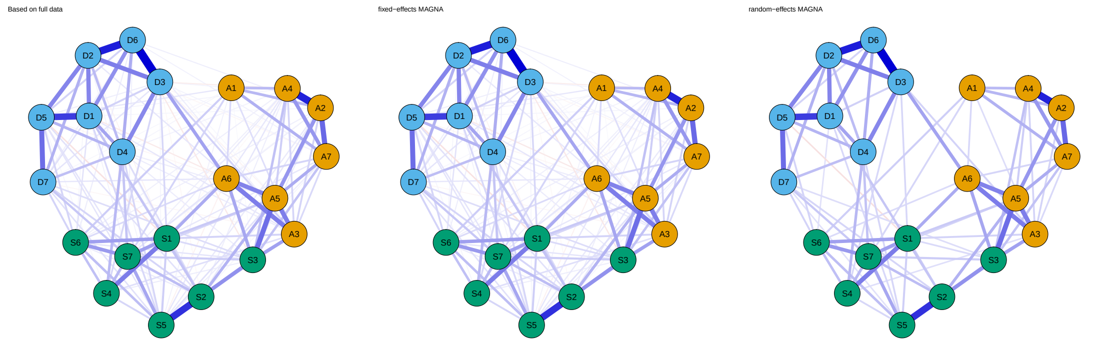

(a) Estimated network structures from the short version of the Depression Anxiety Stress Scales (DASS21; Lovibond and Lovibond, 1995). Orange nodes indicate anxiety items, blue nodes depression items, and green nodes stress items. Node descriptions can be read in Table 1.

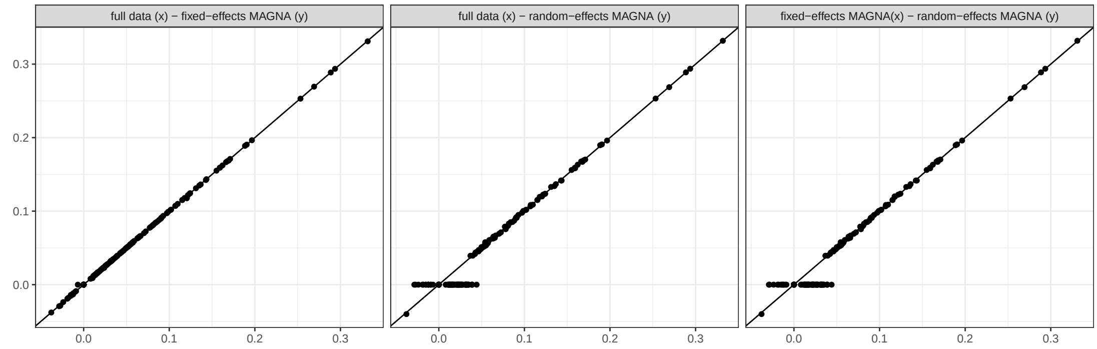

(b) Correspondence of estimated edge weights between different graphs shown in Figure 1a.

Figure 1: Results of the Depression Anxiety Stress Scales (DASS21; Lovibond and Lovibond, 1995) analysis. Three models were estimated: (1) a model on the entire dataset ($n = 39{,}775$), (2) a fixed-effects MAGNA model on the data split in 40 random samples (average $n = 994.3$), and (3) a random-effects MAGNA fitted on the same data split in 40 samples.

Edge weights that were included in the random-effects GGM were near identical to edge weights in the other models (Panel 1b). The average random-effects standard deviation was estimated to be 0.06, which is lower than the PTSD example, but still relatively high. As such, this dataset shows that all methods recover the same dominant structure, but also that including random effects in the model costs statistical power in detecting weaker edge weights, which is not surprising given the vastly increased model complexity of random-effects MAGNA compared to fixed-effects MAGNA.

5 Estimating Multi-group Ising Models from Summary Statistics

While the current paper focuses on the GGM for continuous data, there are other network models for other types of data that are also commonly used. The most prevalent other network model used is the *Ising Model* (Epskamp, Maris, et al., 2018; Ising, 1925; Marsman et al., 2018), which models dichotomous data. Originally used to model ferromagnetism, A particularly interesting property of the Ising model is that it can feature two stable states rather than one as in the GGM (Dalege et al., 2016; Dalege et al., 2018). More importantly, this behavior can be modeled with a single parameter, β, that controls the *temperature* in a system. Common methods for estimating Ising model parameters from data, such as the *eLasso* algorithm (van Borkulo et al., 2014), cannot directly estimate the β parameter, as it is not identified together with the network structure. We can make use of the estimator presented in Section 2 of the main text to also estimate Ising models together with equality constrains across parameters as well as across multiple datasets. In addition, we will see that these estimators also allow for the estimation of Ising model parameters from summary statistics—the variance–covariance matrix of the data—a novel contribution which has not yet been implemented in user-friendly software. The presented methods below have been implemented in the *psychonetrics* (Epskamp, 2020b) package, and can be used through the function `Ising`. The package also contains example code and an example dataset.

Let $\boldsymbol{y}_C$ now represent a dichotomous random variable vector with observation $\boldsymbol{y}_c \in \{a, b\}^p$ for case $c \in 1, \ldots, n$. Typically, encoding $a = 0$ and $b = 1$ are used in psychometric modeling and encodings $a = -1$ and $b = 1$ are used when using the Ising model as a computational model. While in the single group setting these encodings are equivalent and resulting parameters can be transformed between the two encodings (Epskamp, Maris, et al., 2018), they are not equivalent in the multi-group setting with equality constrains on some parameters across groups. The interpretation of some parameters can be vastly different as well depending on the encoding used (Haslbeck et al., 2020). As such, this is an important consideration to make before estimating an Ising model. For the expressions below, however, the encoding is irrelevant, and can arbitrarily be defined as a and b.

The Ising model can be characterized using the following probability function:

$$\Pr(\boldsymbol{y}_C = \boldsymbol{y}_c) = \frac{\exp\left(-\beta H\left(\boldsymbol{y}_c; \boldsymbol{\tau}, \boldsymbol{\Omega}\right)\right)}{Z(\boldsymbol{\tau}, \boldsymbol{\Omega})}.$$

With $\boldsymbol{\tau}$ representing a set of intercepts (also termed 'thresholds' or 'the external field') and $\boldsymbol{\Omega}$ representing the network structure. Here, $H\left(\boldsymbol{y}_c; \boldsymbol{\tau}, \boldsymbol{\Omega}\right)$ represents the *Hamiltonian* potential function, which is a function of the elements of $\boldsymbol{\tau}$ and $\boldsymbol{\Omega}$ (simplifying notation by dropping subscript c):

$$H\left(\boldsymbol{y}; \boldsymbol{\tau}, \boldsymbol{\Omega}\right) = -\sum_{i=1}^{p} \tau_i y_i - \sum_{i=2}^{p} \sum_{j=1}^{i-1} \omega_{ij} y_i y_j.$$

The function $Z(\boldsymbol{\tau}, \boldsymbol{\Omega})$ is a normalizing constant with respect to the data, also called the *partition function*, which sums over all possible outcomes for $\boldsymbol{y}_C$:

$$Z(\boldsymbol{\tau}, \boldsymbol{\Omega}) = \sum_{\boldsymbol{y}} \exp\left(-\beta H\left(\boldsymbol{y}; \boldsymbol{\tau}, \boldsymbol{\Omega}\right)\right).$$

The notation $\sum_{\boldsymbol{y}}$ here indicates that a sum is taken over all possible outcomes for $\boldsymbol{y}_C$. This sum can be very large, and becomes computationally intractable for models with many (e.g., over 20) nodes. However, many applications of Ising models in psychological datasets are on network structures of fewer nodes (e.g., Fried et al., 2015), in which case the evaluation of Z is entirely feasible.

Let $v_i^{(1)} = \sum_{c=1}^{n} \left(y_{ci}\right)$ and $v_{ij}^{(2)} = \sum_{c=1}^{n} \left(y_{ci} y_{cj}\right)$ represent *summary statistics* of the data. These summary statistics can also be obtained directly from the sample means and sample variance–covariance matrix discussed in Section of the main text (there presented as sample means and covariances of the sample correlations rather than of the raw data):

$$\begin{aligned}
\boldsymbol{v}^{(1)} &= n\bar{\boldsymbol{y}} \\
\boldsymbol{V}^{(2)} &= n\left(\bar{\boldsymbol{S}} + \bar{\boldsymbol{y}}\bar{\boldsymbol{y}}^{\top}\right).
\end{aligned}$$

Using these summary statistics, we can characterize the sum of Hamiltonians over all cases, here defined as H^*, as a function of these summary statistics:

$$H^*\left(\boldsymbol{v}^{(1)}, \boldsymbol{V}^{(2)}; \boldsymbol{\tau}, \boldsymbol{\Omega}\right) = \sum_{p=1}^{n} H\left(\boldsymbol{y}_p; \boldsymbol{\tau}, \boldsymbol{\Omega}\right) = -\sum_{i=1}^{m} \tau_i v_i^{(1)} - \sum_{i=2}^{m} \sum_{j=1}^{i-1} \omega_{ij} v_{ij}^{(2)}.$$

The fit function, $-2/n$ times the log likelihood, then becomes:

$$F_{\text{ml}} = 2\ln\left(Z(\boldsymbol{\tau}, \boldsymbol{\Omega})\right) + \frac{2\beta}{n} H^*\left(\boldsymbol{v}^{(1)}, \boldsymbol{V}^{(2)}; \boldsymbol{\tau}, \boldsymbol{\Omega}\right).$$

In this setting, we will not further model the parameters of the Ising model. As such, the distributional parameters $\boldsymbol{\phi}$ and the model parameters $\boldsymbol{\theta}$ are the same. For the single-group setting, these becomes:

$$\boldsymbol{\phi} = \boldsymbol{\theta} = \begin{bmatrix} \boldsymbol{\tau} \\ \boldsymbol{\omega} \\ \beta \end{bmatrix},$$

in which $\boldsymbol{\omega} = \text{vechs}\left(\boldsymbol{\Omega}\right)$. This also simplifies the model Jacobian to be:

$$\frac{\partial \boldsymbol{\phi}}{\partial \boldsymbol{\theta}} = \boldsymbol{I}.$$

Other setups are possible. For example, one could aim to model a low-rank Ising model as described by Marsman et al. (2015) in this setting as well by assigning a different model Jacobian. The manual Jacobian will be a matrix of ones and zeroes indicating which parameters are free to be estimated and which are fixed to their starting values. As the inverse temperature parameter β is not identified together with $\boldsymbol{\tau}$ and $\boldsymbol{\Omega}$, it is typically constrained to 1, which can be modeled with:

$$\frac{\partial \boldsymbol{\theta}}{\partial \boldsymbol{\phi}} = \begin{bmatrix} \boldsymbol{I} & \boldsymbol{O} & \boldsymbol{0} \\ \boldsymbol{O} & \boldsymbol{I} & \boldsymbol{0} \\ \boldsymbol{0}^\top & \boldsymbol{0}^\top & 0 \end{bmatrix},$$

which is an identity matrix with the last diagonal element set to zero. Other (equality) constrains in the manual Jacobian can identify β, especially when extended to the multi-group setting.

The distribution Jacobian for the single-group Ising model takes the following form:

$$\frac{\partial F}{\partial \boldsymbol{\psi}} = \begin{bmatrix} \frac{\partial F}{\partial \boldsymbol{\tau}} & \frac{\partial F}{\partial \boldsymbol{\omega}} & \frac{\partial F}{\partial \beta} \end{bmatrix},$$

which is a row vector with each element indicating the derivative of the fit function to that particular parameter. It can be derived that these derivatives follow the difference between observed and expected summary statistics:

$$\begin{aligned} \frac{\partial F}{\partial \tau_i} &= 2\beta \mathcal{E}\left(y_i\right) - 2\beta \frac{v_i^{(1)}}{n} \\ \frac{\partial F}{\partial \omega_{ij}} &= 2\beta \mathcal{E}\left(y_i y_j\right) - 2\beta \frac{v_{ij}^{(2)}}{n} \\ \frac{\partial F}{\partial \beta} &= 2\frac{H^*}{n} - 2\mathcal{E}\left(H\right). \end{aligned}$$

Let $\boldsymbol{H}_{\dots}$ represent an element of the distribution Hessian:

$$\mathcal{E}\left[\frac{\partial \boldsymbol{\nabla} F}{\partial \boldsymbol{\phi}}\right] = \begin{bmatrix} \boldsymbol{H}_{\tau_1,\tau_1} & \boldsymbol{H}_{\tau_1,\tau_2} & \dots & \boldsymbol{H}_{\tau_1,\omega_{11}} & \boldsymbol{H}_{\tau_1,\omega_{21}} & \dots & \boldsymbol{H}_{\tau_1,\beta} \\ \boldsymbol{H}_{\tau_2,\tau_1} & \boldsymbol{H}_{\tau_2,\tau_2} & \dots & \boldsymbol{H}_{\tau_2,\omega_{11}} & \boldsymbol{H}_{\tau_2,\omega_{21}} & \dots & \boldsymbol{H}_{\tau_2,\beta} \\ \vdots & \vdots & \vdots & \vdots & \vdots & \vdots & \vdots \\ \boldsymbol{H}_{\omega_{11},\tau_1} & \boldsymbol{H}_{\omega_{11},\tau_2} & \dots & \boldsymbol{H}_{\omega_{11},\omega_{11}} & \boldsymbol{H}_{\omega_{11},\omega_{21}} & \dots & \boldsymbol{H}_{\omega_{11},\beta} \\ \boldsymbol{H}_{\omega_{21},\tau_1} & \boldsymbol{H}_{\omega_{21},\tau_2} & \dots & \boldsymbol{H}_{\omega_{21},\omega_{11}} & \boldsymbol{H}_{\omega_{21},\omega_{21}} & \dots & \boldsymbol{H}_{\omega_{21},\beta} \\ \vdots & \vdots & \vdots & \vdots & \vdots & \vdots & \vdots \\ \boldsymbol{H}_{\beta,\tau_1} & \boldsymbol{H}_{\beta,\tau_2} & \dots & \boldsymbol{H}_{\beta,\omega_{11}} & \boldsymbol{H}_{\beta,\omega_{21}} & \dots & \boldsymbol{H}_{\beta,\beta} \end{bmatrix}.$$

These elements then take the following form:

$$
\begin{aligned}
\boldsymbol{H}_{\tau_i,\tau_j} &= 2\beta^2 \left(\mathcal{E}\left(y_i y_j\right) - \mathcal{E}\left(y_i\right)\mathcal{E}(y_j)\right)\\
\boldsymbol{H}_{\omega_{ij},\tau_k} &= 2\beta^2 \left(\mathcal{E}\left(y_i y_j y_k\right) - \mathcal{E}\left(y_i y_j\right)\mathcal{E}\left(y_k\right)\right)\\
\boldsymbol{H}_{\omega_{ij},\omega_{kl}} &= 2\beta^2 \left(\mathcal{E}\left(y_i y_j y_k y_l\right) - \mathcal{E}\left(y_i y_j\right)\mathcal{E}\left(y_k y_l\right)\right)\\
\boldsymbol{H}_{\tau_i,\beta} &= 2\left(\mathcal{E}\left(y_i\right)\mathcal{E}\left(H\right) - \mathcal{E}\left(y_i H\right)\right)\\
\boldsymbol{H}_{\omega_{ij},\beta} &= 2\left(\mathcal{E}\left(y_i y_j\right)\mathcal{E}\left(H\right) - \mathcal{E}\left(y_i y_j H\right)\right)\\
\boldsymbol{H}_{\beta,\beta} &= 2\left(\mathcal{E}\left(H\right)^2 - \mathcal{E}\left(H^2\right)\right).
\end{aligned}
$$

With these expressions, the Ising model can be estimated using maximum likelihood estimation from the sample means and variance–covariance structure exactly in the same manner the GGM can be estimated as described in the main text and supplementary materials. This allows for imposing equality constrains as well as to estimate an Ising model with certain edge weights fixed to zero. Extension to the multi-group setting can also be performed in exactly the same manner as described in the main text: by specifying the distribution Jacobians and Hessians described above for each group and imposing equality constrains across groups in the manual Jacobian.

References

Dalege, J., Borsboom, D., van Harreveld, F., van den Berg, H., Conner, M., & van der Maas, H. L. J. (2016). Toward a formalized account of attitudes: The Causal Attitude Network (CAN) model. *Psychological Review*, *123*(1), 2–22. https://doi.org/10.1037/a0039802

Dalege, J., Borsboom, D., van Harreveld, F., & van der Maas, H. L. (2018). The attitudinal entropy (ae) framework as a general theory of individual attitudes. *Psychological Inquiry*, *29*(4), 175–193. https://doi.org/10.1080/1047840X.2018.1537246

Epskamp, S. (2020a). Psychometric network models from time-series and panel data. *Psychometrika*. https://doi.org/10.1007/s11336-020-09697-3

Epskamp, S. (2020b). *Psychonetrics: Structural equation modeling and confirmatory network analysis* [R package version 0.8]. R package version 0.8. http://psychonetrics.org/

Epskamp, S., Cramer, A. O. J., Waldorp, L. J., Schmittmann, V. D., Borsboom, D., Waldrop, L. J., Schmittmann, V. D., & Borsboom, D. (2012). qgraph : Network Visualizations of Relationships in Psychometric Data. *Journal of Statistical Software*, *48*(4), 1–18. https://doi.org/10.18637/jss.v048.i04

Epskamp, S., Maris, G. K. J., Waldorp, L. J., & Borsboom, D. (2018). Network Psychometrics (P. Irwing, D. Hughes, & T. Booth, Eds.). In P. Irwing, D. Hughes, & T. Booth (Eds.), *Handbook of psychometrics*. New York, NY, USA, Wiley. http://arxiv.org/abs/1609.02818

Epskamp, S., Waldorp, L. J., Mõttus, R., & Borsboom, D. (2018). The Gaussian Graphical Model in Cross-Sectional and Time-Series Data. *Multivariate Behavioral Research*, *53*(4), 453–480. https://doi.org/10.1080/00273171.2018.1454823

Fried, E. I., Bockting, C., Arjadi, R., Borsboom, D., Amshoff, M., Cramer, A. O. J., Epskamp, S., Tuerlinckx, F., Carr, D., & Stroebe, M. (2015). From loss to loneliness: The relationship between bereavement and depressive symptoms. *Journal of abnormal psychology*, *124*(2), 256–265. https://doi.org/10.1037/abn0000028

Friedman, J. H., Hastie, T., & Tibshirani, R. (2019). *Glasso: Graphical lasso: Estimation of gaussian graphical models* [R package version 1.11]. R package version 1.11. https://CRAN.R-project.org/package=glasso

Friedman, J. H., Hastie, T., & Tibshirani, R. (2008). Sparse inverse covariance estimation with the graphical lasso. *Biostatistics*, *9*(3), 432–441. https://doi.org/10.1093/biostatistics/kxm045

Haslbeck, J. M., Epskamp, S., Marsman, M., & Waldorp, L. J. (2020). Interpreting the ising model: The input matters. *Multivariate Behavioral Research*, 1–11. https://doi.org/10.1080/00273171.2020.1730150

Ising, E. (1925). Beitrag zur theorie des ferromagnetismus. *Zeitschrift f{ü}r Physik A Hadrons and Nuclei*, *31*(1), 253–258. https://doi.org/10.1007/BF02980577

Lauritzen, S. L. (1996). *Graphical Models*. Oxford, UK, Clarendon Press.

Lovibond, P. F., & Lovibond, S. H. (1995). The structure of negative emotional states: Comparison of the depression anxiety stress scales (dass) with the beck depression and anxiety inventories. *Behaviour research and therapy*, *33*(3), 335–343.

Marsman, M., Borsboom, D., Kruis, J., Epskamp, S., van Bork, R., Waldorp, L., Maas, H., Maris, G., Bork, V., Waldorp, L., Van Der Maas, H. L. J., Maris, G., & Marsman, M. (2018). An Introduction to Network Psychometrics: Relating Ising Network Models to Item Response Theory Models. *Multivariate Behavioral Research*, *53*(1), 15–35. https://doi.org/10.1080/00273171.2017.1379379

Marsman, M., Maris, G., Bechger, T., & Glas, C. (2015). Bayesian inference for low-rank Ising networks. *Scientific reports*, *5*(9050), 1–7. https://doi.org/10.1038/srep09050

Mohammadi, R., & Wit, E. C. (2019). BDgraph: An R package for Bayesian structure learning in graphical models. *Journal of Statistical Software*, *89*(3), 1–30. https://doi.org/10.18637/jss.v089.i03

R Core Team. (2019). *R: A language and environment for statistical computing*. R Foundation for Statistical Computing. Vienna, Austria. https://www.R-project.org/

Schafer, J., Opgen-Rhein, R., Zuber, V., Ahdesmaki, M., Silva, A. P. D., & Strimmer., K. (2017). *Corpcor: Efficient estimation of covariance and (partial) correlation* [R package version 1.6.9]. R package version 1.6.9. https://CRAN.R-project.org/package=corpcor

van Borkulo, C. D., Borsboom, D., Epskamp, S., Blanken, T. F., Boschloo, L., Schoevers, R. A., & Waldorp, L. J. (2014). A new method for constructing networks from binary data. *Nature: Scientific Reports*, *4*(5918). https://doi.org/10.1038/srep05918

Wickham, H., François, R., Henry, L., & Müller, K. (2019). *Dplyr: A grammar of data manipulation* [R package version 0.8.3]. R package version 0.8.3. https://CRAN.R-project.org/package=dplyr

Williams, D. R., & Mulder, J. (2020). Bayesian hypothesis testing for gaussian graphical models: Conditional independence and order constraints. *Journal of Mathematical Psychology*, *99*, 102441. https://doi.org/10.1016/j.jmp.2020.102441

Williams, D. R., Rhemtulla, M., Wysocki, A. C., & Rast, P. (2019). On nonregularized estimation of psychological networks. *Multivariate behavioral research*, *54*(5), 719–750. https://doi.org/10.1080/00273171.2019.1575716
